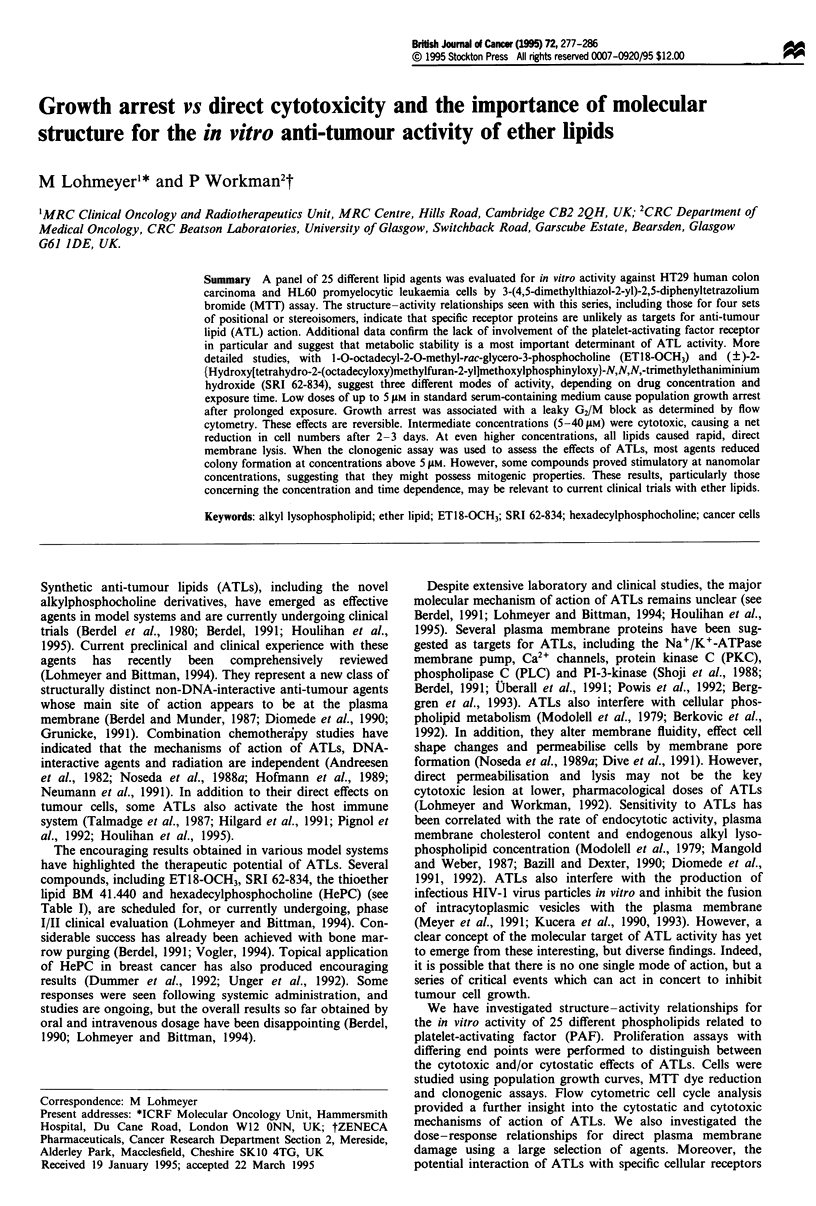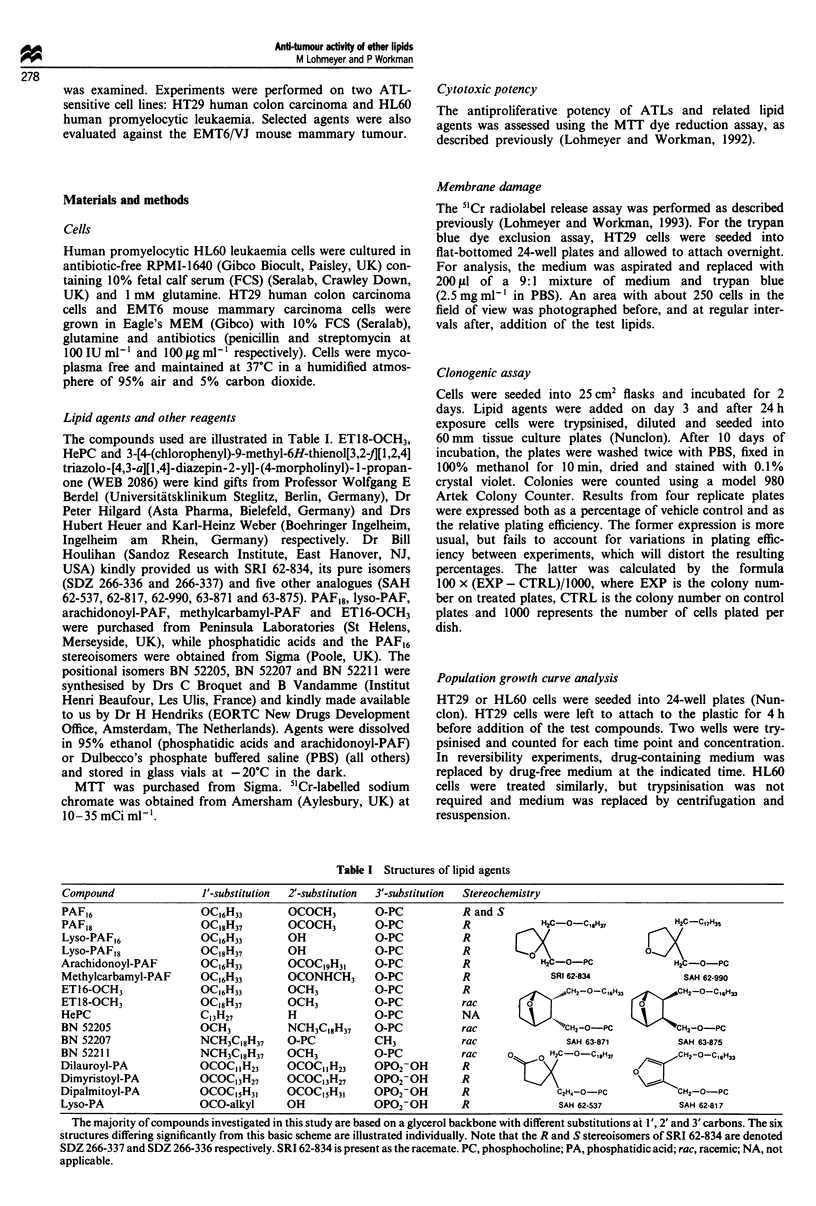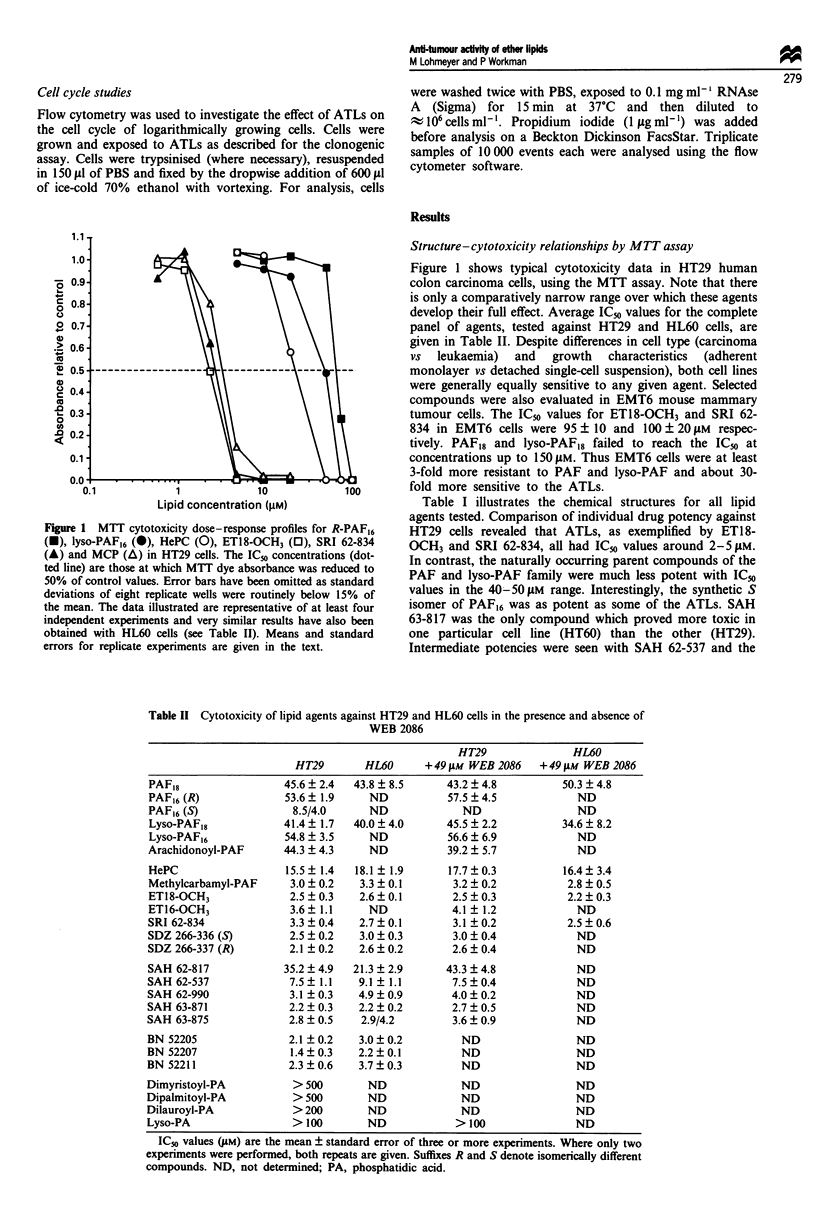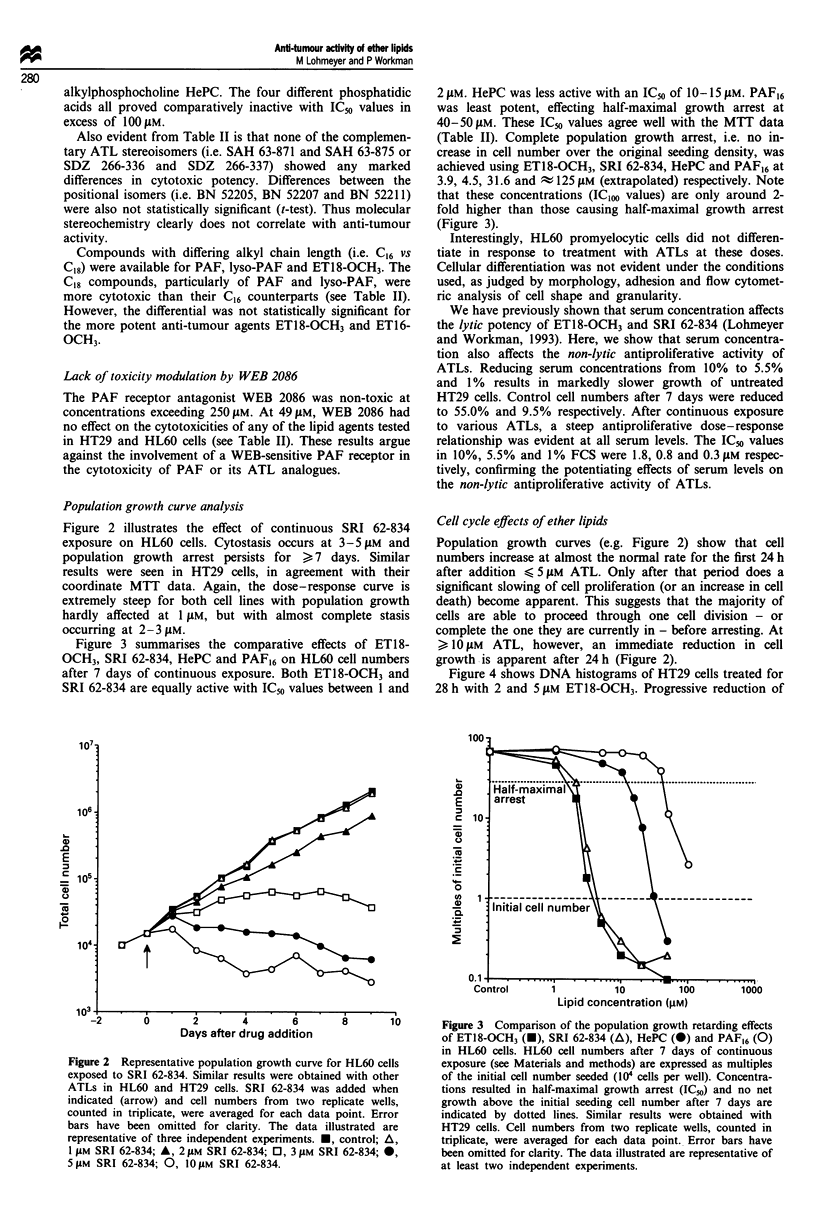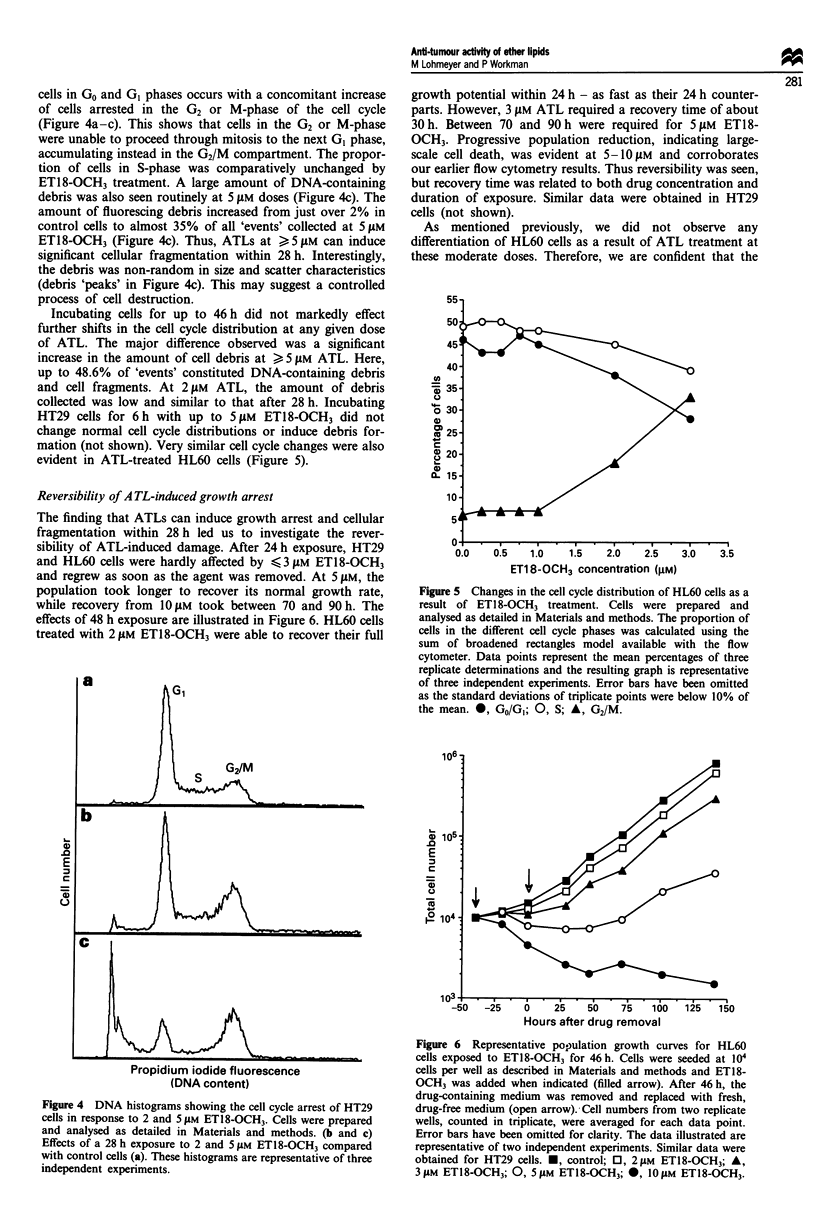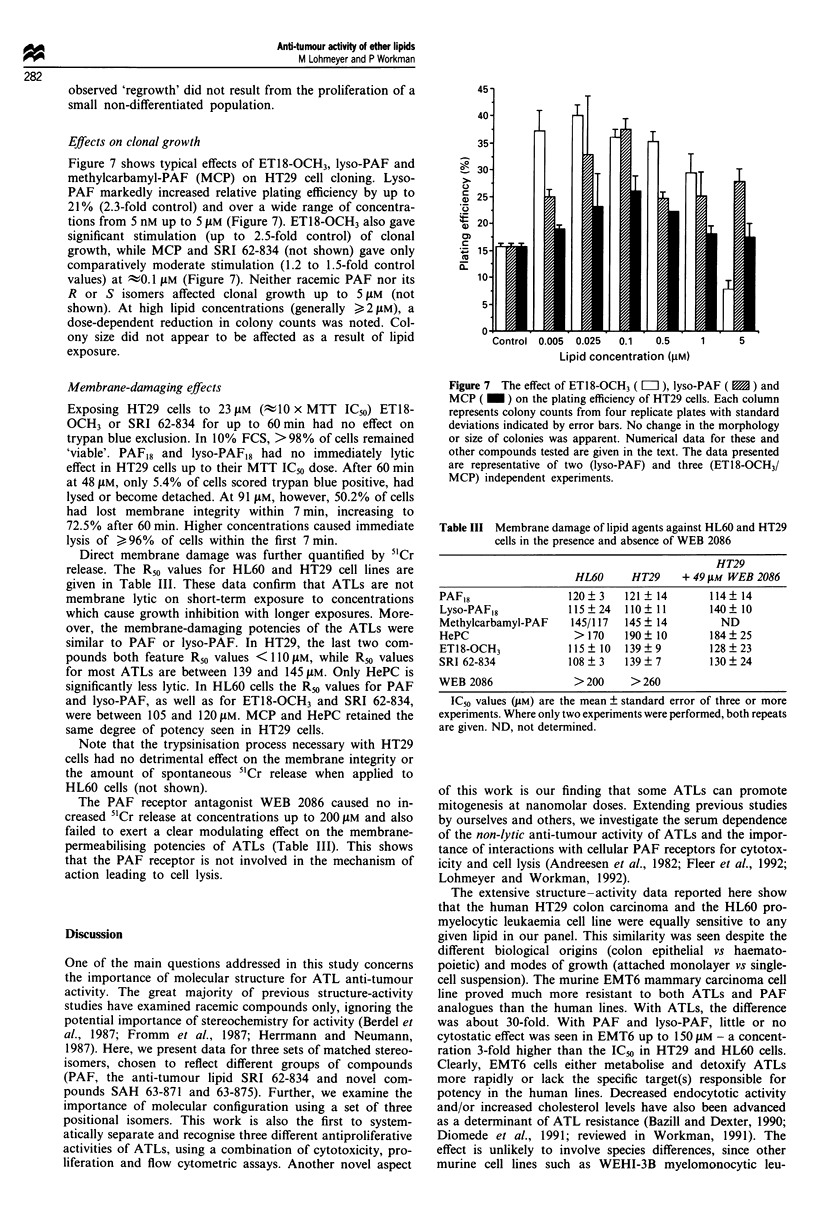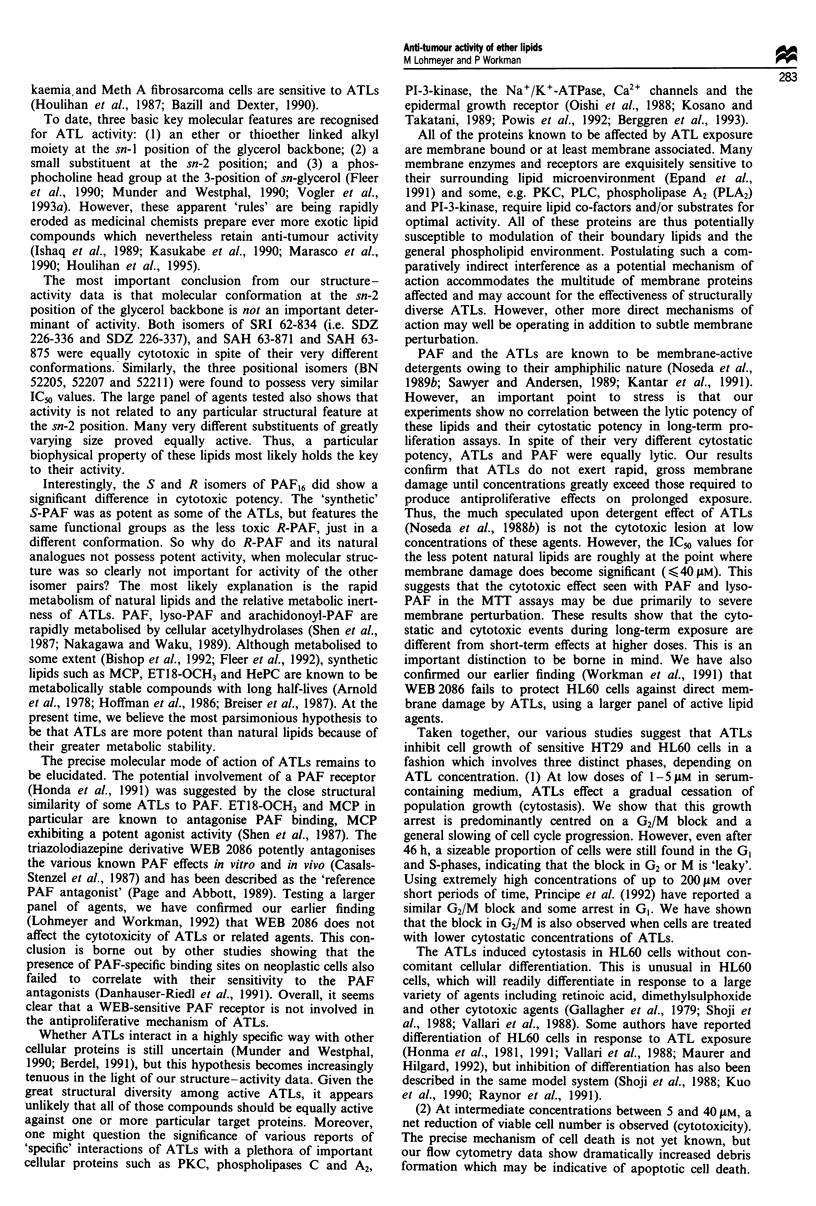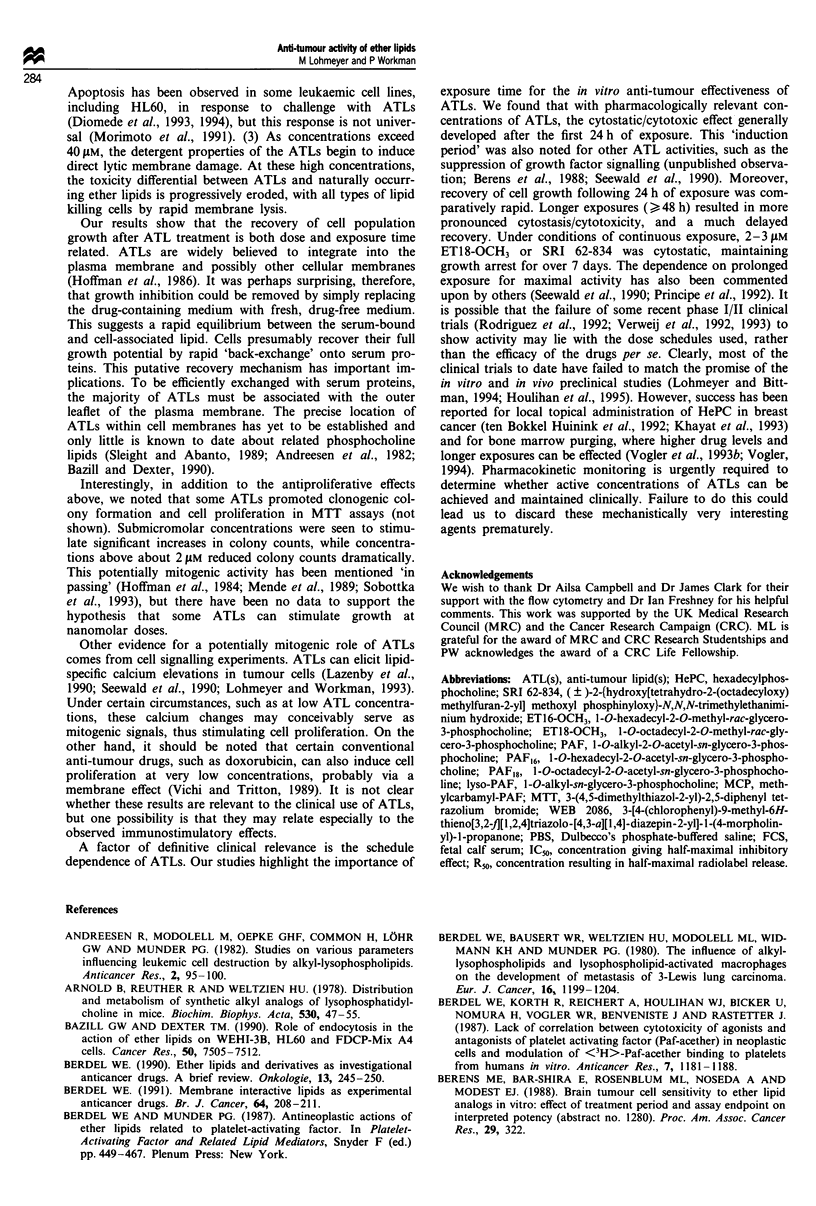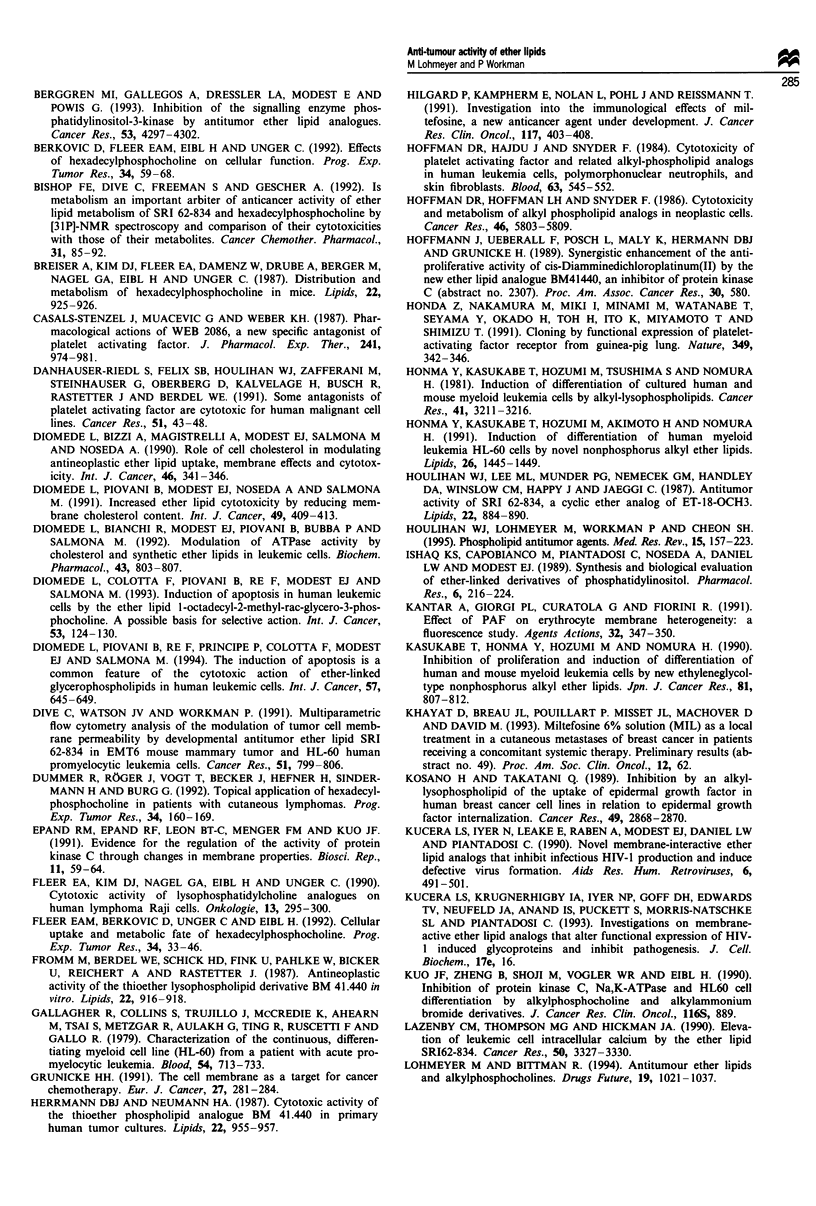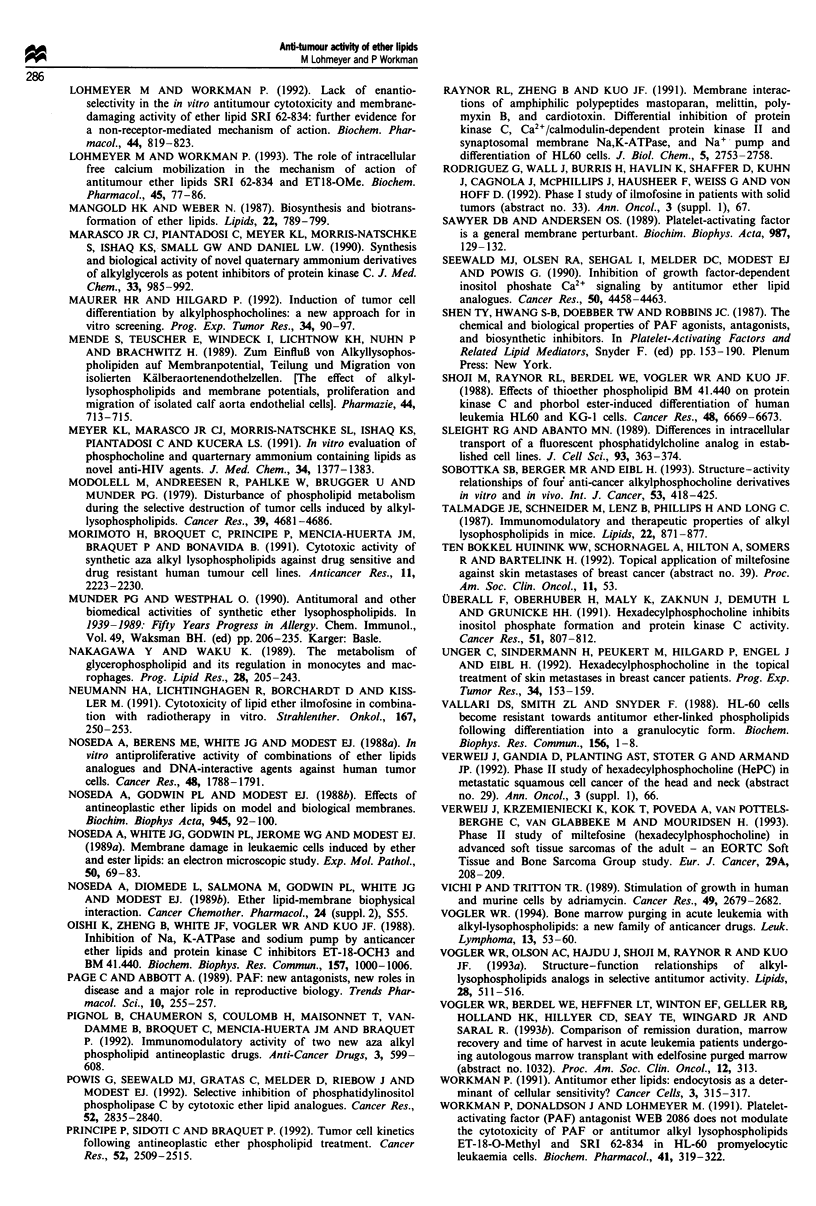# Growth arrest vs direct cytotoxicity and the importance of molecular structure for the in vitro anti-tumour activity of ether lipids.

**DOI:** 10.1038/bjc.1995.325

**Published:** 1995-08

**Authors:** M. Lohmeyer, P. Workman

**Affiliations:** MRC Clinical Oncology and Radiotherapeutics Unit, MRC Centre, Cambridge, UK.

## Abstract

A panel of 25 different lipid agents was evaluated for in vitro activity against HT29 human colon carcinoma and HL60 promyelocytic leukaemia cells by 3-(4,5-dimethylthiazol-2-yl)-2,5-diphenyltetrazolium bromide (MTT) assay. The structure-activity relationships seen with this series, including those for four sets of positional or stereoisomers, indicate that specific receptor proteins are unlikely as targets for anti-tumour lipid (ATL) action. Additional data confirm the lack of involvement of the platelet-activating factor receptor in particular and suggest that metabolic stability is a most important determinant of ATL activity. More detailed studies, with 1-O-octadecyl-2-O-methyl-rac-glycero-3-phosphocholine (ET18-OCH3) and (+/-)-2-(Hydroxy[tetrahydro-2-(octadecyloxy)methylfuran-2- yl]methoxyphosphinyloxy)-N,N,N,-trimethylethaniminium hydroxide (SRI 62-834), suggest three different modes of activity, depending on drug concentration and exposure time. Low doses of up to 5 microM in standard serum-containing medium cause population growth arrest after prolonged exposure. Growth arrest was associated with a leaky G2/M block as determined by flow cytometry. These effects are reversible. Intermediate concentrations (5-40 microM) were cytotoxic, causing a net reduction in cell numbers after 2-3 days. At even higher concentrations, all lipids caused rapid, direct membrane lysis. When the clonogenic assay was used to assess the effects of ATLs, most agents reduced colony formation at concentrations above 5 microM. However, some compounds proved stimulatory at nanomolar concentrations, suggesting that they might possess mitogenic properties. These results, particularly those concerning the concentration and time dependence, may be relevant to current clinical trials with ether lipids.


British Journal of Cancer (1995) 72, 277-286
© 1995 Stockton Press All rights reserved 0007-0920/95 $12.00

### Growth arrest *vs* direct cytotoxicity and the importance of molecular structure for the *in vitro* anti-tumour activity of ether lipids

M Lohmeyer[1]* and P Workman[2]†

[1]*MRC Clinical Oncology and Radiotherapeutics Unit, MRC Centre, Hills Road, Cambridge CB2 2QH, UK;* [2]*CRC Department of Medical Oncology, CRC Beatson Laboratories, University of Glasgow, Switchback Road, Garscube Estate, Bearsden, Glasgow G61 1DE, UK.*

**Summary** A panel of 25 different lipid agents was evaluated for *in vitro* activity against HT29 human colon carcinoma and HL60 promyelocytic leukaemia cells by 3-(4,5-dimethylthiazol-2-yl)-2,5-diphenyltetrazolium bromide (MTT) assay. The structure–activity relationships seen with this series, including those for four sets of positional or stereoisomers, indicate that specific receptor proteins are unlikely as targets for anti-tumour lipid (ATL) action. Additional data confirm the lack of involvement of the platelet-activating factor receptor in particular and suggest that metabolic stability is a most important determinant of ATL activity. More detailed studies, with 1-O-octadecyl-2-O-methyl-*rac*-glycero-3-phosphocholine (ET18-$OCH_3$) and (±)-2-{Hydroxy[tetrahydro-2-(octadecyloxy)methylfuran-2-yl]methoxylphosphinyloxy}-*N,N,N*,-trimethylethaniminium hydroxide (SRI 62-834), suggest three different modes of activity, depending on drug concentration and exposure time. Low doses of up to 5 μM in standard serum-containing medium cause population growth arrest after prolonged exposure. Growth arrest was associated with a leaky $G_2$/M block as determined by flow cytometry. These effects are reversible. Intermediate concentrations (5–40 μM) were cytotoxic, causing a net reduction in cell numbers after 2–3 days. At even higher concentrations, all lipids caused rapid, direct membrane lysis. When the clonogenic assay was used to assess the effects of ATLs, most agents reduced colony formation at concentrations above 5 μM. However, some compounds proved stimulatory at nanomolar concentrations, suggesting that they might possess mitogenic properties. These results, particularly those concerning the concentration and time dependence, may be relevant to current clinical trials with ether lipids.

**Keywords**: alkyl lysophospholipid; ether lipid; ET18-$OCH_3$; SRI 62-834; hexadecylphosphocholine; cancer cells

Synthetic anti-tumour lipids (ATLs), including the novel alkylphosphocholine derivatives, have emerged as effective agents in model systems and are currently undergoing clinical trials (Berdel *et al.*, 1980; Berdel, 1991; Houlihan *et al.*, 1995). Current preclinical and clinical experience with these agents has recently been comprehensively reviewed (Lohmeyer and Bittman, 1994). They represent a new class of structurally distinct non-DNA-interactive anti-tumour agents whose main site of action appears to be at the plasma membrane (Berdel and Munder, 1987; Diomede *et al.*, 1990; Grunicke, 1991). Combination chemotherapy studies have indicated that the mechanisms of action of ATLs, DNA-interactive agents and radiation are independent (Andreesen *et al.*, 1982; Noseda *et al.*, 1988*a*; Hofmann *et al.*, 1989; Neumann *et al.*, 1991). In addition to their direct effects on tumour cells, some ATLs also activate the host immune system (Talmadge *et al.*, 1987; Hilgard *et al.*, 1991; Pignol *et al.*, 1992; Houlihan *et al.*, 1995).

The encouraging results obtained in various model systems have highlighted the therapeutic potential of ATLs. Several compounds, including ET18-$OCH_3$, SRI 62-834, the thioether lipid BM 41.440 and hexadecylphosphocholine (HePC) (see Table I), are scheduled for, or currently undergoing, phase I/II clinical evaluation (Lohmeyer and Bittman, 1994). Considerable success has already been achieved with bone marrow purging (Berdel, 1991; Vogler, 1994). Topical application of HePC in breast cancer has also produced encouraging results (Dummer *et al.*, 1992; Unger *et al.*, 1992). Some responses were seen following systemic administration, and studies are ongoing, but the overall results so far obtained by oral and intravenous dosage have been disappointing (Berdel, 1990; Lohmeyer and Bittman, 1994).

Correspondence: M Lohmeyer
Present addresses: *ICRF Molecular Oncology Unit, Hammersmith Hospital, Du Cane Road, London W12 0NN, UK; †ZENECA Pharmaceuticals, Cancer Research Department Section 2, Mereside, Alderley Park, Macclesfield, Cheshire SK10 4TG, UK
Received 19 January 1995; accepted 22 March 1995

Despite extensive laboratory and clinical studies, the major molecular mechanism of action of ATLs remains unclear (see Berdel, 1991; Lohmeyer and Bittman, 1994; Houlihan *et al.*, 1995). Several plasma membrane proteins have been suggested as targets for ATLs, including the $Na^+/K^+$-ATPase membrane pump, $Ca^{2+}$ channels, protein kinase C (PKC), phospholipase C (PLC) and PI-3-kinase (Shoji *et al.*, 1988; Berdel, 1991; Überall *et al.*, 1991; Powis *et al.*, 1992; Berggren *et al.*, 1993). ATLs also interfere with cellular phospholipid metabolism (Modolell *et al.*, 1979; Berkovic *et al.*, 1992). In addition, they alter membrane fluidity, effect cell shape changes and permeabilise cells by membrane pore formation (Noseda *et al.*, 1989*a*; Dive *et al.*, 1991). However, direct permeabilisation and lysis may not be the key cytotoxic lesion at lower, pharmacological doses of ATLs (Lohmeyer and Workman, 1992). Sensitivity to ATLs has been correlated with the rate of endocytotic activity, plasma membrane cholesterol content and endogenous alkyl lysophospholipid concentration (Modolell *et al.*, 1979; Mangold and Weber, 1987; Bazill and Dexter, 1990; Diomede *et al.*, 1991, 1992). ATLs also interfere with the production of infectious HIV-1 virus particles *in vitro* and inhibit the fusion of intracytoplasmic vesicles with the plasma membrane (Meyer *et al.*, 1991; Kucera *et al.*, 1990, 1993). However, a clear concept of the molecular target of ATL activity has yet to emerge from these interesting, but diverse findings. Indeed, it is possible that there is no one single mode of action, but a series of critical events which can act in concert to inhibit tumour cell growth.

We have investigated structure–activity relationships for the *in vitro* activity of 25 different phospholipids related to platelet-activating factor (PAF). Proliferation assays with differing end points were performed to distinguish between the cytotoxic and/or cytostatic effects of ATLs. Cells were studied using population growth curves, MTT dye reduction and clonogenic assays. Flow cytometric cell cycle analysis provided a further insight into the cytostatic and cytotoxic mechanisms of action of ATLs. We also investigated the dose–response relationships for direct plasma membrane damage using a large selection of agents. Moreover, the potential interaction of ATLs with specific cellular receptors was examined. Experiments were performed on two ATL-sensitive cell lines: HT29 human colon carcinoma and HL60 human promyelocytic leukaemia. Selected agents were also evaluated against the EMT6/VJ mouse mammary tumour.

### Materials and methods

### *Cells*

Human promyelocytic HL60 leukaemia cells were cultured in antibiotic-free RPMI-1640 (Gibco Biocult, Paisley, UK) containing 10% fetal calf serum (FCS) (Seralab, Crawley Down, UK) and 1 mM glutamine. HT29 human colon carcinoma cells and EMT6 mouse mammary carcinoma cells were grown in Eagle's MEM (Gibco) with 10% FCS (Seralab), glutamine and antibiotics (penicillin and streptomycin at 100 IU $ml^{-1}$ and 100 $\mu g\ ml^{-1}$ respectively). Cells were mycoplasma free and maintained at 37°C in a humidified atmosphere of 95% air and 5% carbon dioxide.

### *Lipid agents and other reagents*

The compounds used are illustrated in Table I. ET18-$OCH_3$, HePC and 3-[4-(chlorophenyl)-9-methyl-6*H*-thienol[3,2-*f*][1,2,4] triazolo-[4,3-*a*][1,4]-diazepin-2-yl]-(4-morpholinyl)-1-propanone (WEB 2086) were kind gifts from Professor Wolfgang E Berdel (Universitätsklinikum Steglitz, Berlin, Germany), Dr Peter Hilgard (Asta Pharma, Bielefeld, Germany) and Drs Hubert Heuer and Karl-Heinz Weber (Boehringer Ingelheim, Ingelheim am Rhein, Germany) respectively. Dr Bill Houlihan (Sandoz Research Institute, East Hanover, NJ, USA) kindly provided us with SRI 62-834, its pure isomers (SDZ 266-336 and 266-337) and five other analogues (SAH 62-537, 62-817, 62-990, 63-871 and 63-875). $PAF_{18}$, lyso-PAF, arachidonoyl-PAF, methylcarbamyl-PAF and ET16-$OCH_3$ were purchased from Peninsula Laboratories (St Helens, Merseyside, UK), while phosphatidic acids and the $PAF_{16}$ stereoisomers were obtained from Sigma (Poole, UK). The positional isomers BN 52205, BN 52207 and BN 52211 were synthesised by Drs C Broquet and B Vandamme (Institut Henri Beaufour, Les Ulis, France) and kindly made available to us by Dr H Hendriks (EORTC New Drugs Development Office, Amsterdam, The Netherlands). Agents were dissolved in 95% ethanol (phosphatidic acids and arachidonoyl-PAF) or Dulbecco's phosphate buffered saline (PBS) (all others) and stored in glass vials at −20°C in the dark.

MTT was purchased from Sigma. $^{51}Cr$-labelled sodium chromate was obtained from Amersham (Aylesbury, UK) at 10–35 mCi $ml^{-1}$.

### *Cytotoxic potency*

The antiproliferative potency of ATLs and related lipid agents was assessed using the MTT dye reduction assay, as described previously (Lohmeyer and Workman, 1992).

### *Membrane damage*

The $^{51}Cr$ radiolabel release assay was performed as described previously (Lohmeyer and Workman, 1993). For the trypan blue dye exclusion assay, HT29 cells were seeded into flat-bottomed 24-well plates and allowed to attach overnight. For analysis, the medium was aspirated and replaced with 200 $\mu l$ of a 9:1 mixture of medium and trypan blue (2.5 mg $ml^{-1}$ in PBS). An area with about 250 cells in the field of view was photographed before, and at regular intervals after, addition of the test lipids.

### *Clonogenic assay*

Cells were seeded into 25 $cm^2$ flasks and incubated for 2 days. Lipid agents were added on day 3 and after 24 h exposure cells were trypsinised, diluted and seeded into 60 mm tissue culture plates (Nunclon). After 10 days of incubation, the plates were washed twice with PBS, fixed in 100% methanol for 10 min, dried and stained with 0.1% crystal violet. Colonies were counted using a model 980 Artek Colony Counter. Results from four replicate plates were expressed both as a percentage of vehicle control and as the relative plating efficiency. The former expression is more usual, but fails to account for variations in plating efficiency between experiments, which will distort the resulting percentages. The latter was calculated by the formula 100 × (EXP − CTRL)/1000, where EXP is the colony number on treated plates, CTRL is the colony number on control plates and 1000 represents the number of cells plated per dish.

### *Population growth curve analysis*

HT29 or HL60 cells were seeded into 24-well plates (Nunclon). HT29 cells were left to attach to the plastic for 4 h before addition of the test compounds. Two wells were trypsinised and counted for each time point and concentration. In reversibility experiments, drug-containing medium was replaced by drug-free medium at the indicated time. HL60 cells were treated similarly, but trypsinisation was not required and medium was replaced by centrifugation and resuspension.

**Table I** Structures of lipid agents

| *Compound* | *1′-substitution* | *2′-substitution* | *3′-substitution* | *Stereochemistry* |
|---|---|---|---|---|
| $PAF_{16}$ | $OC_{16}H_{33}$ | $OCOCH_3$ | O-PC | *R* and *S* |
| $PAF_{18}$ | $OC_{18}H_{37}$ | $OCOCH_3$ | O-PC | *R* |
| Lyso-$PAF_{16}$ | $OC_{16}H_{33}$ | OH | O-PC | *R* |
| Lyso-$PAF_{18}$ | $OC_{18}H_{37}$ | OH | O-PC | *R* |
| Arachidonoyl-PAF | $OC_{16}H_{33}$ | $OCOC_{19}H_{31}$ | O-PC | *R* |
| Methylcarbamyl-PAF | $OC_{16}H_{33}$ | $OCONHCH_3$ | O-PC | *R* |
| ET16-$OCH_3$ | $OC_{16}H_{33}$ | $OCH_3$ | O-PC | *R* |
| ET18-$OCH_3$ | $OC_{18}H_{37}$ | $OCH_3$ | O-PC | *rac* |
| HePC | $C_{13}H_{27}$ | H | O-PC | NA |
| BN 52205 | $OCH_3$ | $NCH_3C_{18}H_{37}$ | O-PC | *rac* |
| BN 52207 | $NCH_3C_{18}H_{37}$ | O-PC | $CH_3$ | *rac* |
| BN 52211 | $NCH_3C_{18}H_{37}$ | $OCH_3$ | O-PC | *rac* |
| Dilauroyl-PA | $OCOC_{11}H_{23}$ | $OCOC_{11}H_{23}$ | $OPO_2^-OH$ | *R* |
| Dimyristoyl-PA | $OCOC_{13}H_{27}$ | $OCOC_{13}H_{27}$ | $OPO_2^-OH$ | *R* |
| Dipalmitoyl-PA | $OCOC_{15}H_{31}$ | $OCOC_{15}H_{31}$ | $OPO_2^-OH$ | *R* |
| Lyso-PA | OCO-alkyl | OH | $OPO_2^-OH$ | *R* |

The majority of compounds investigated in this study are based on a glycerol backbone with different substitutions at 1′, 2′ and 3′ carbons. The six structures differing significantly from this basic scheme are illustrated individually. Note that the *R* and *S* stereoisomers of SRI 62-834 are denoted SDZ 266-337 and SDZ 266-336 respectively. SRI 62-834 is present as the racemate. PC, phosphocholine; PA, phosphatidic acid; *rac*, racemic; NA, not applicable.

*Cell cycle studies*

Flow cytometry was used to investigate the effect of ATLs on the cell cycle of logarithmically growing cells. Cells were grown and exposed to ATLs as described for the clonogenic assay. Cells were trypsinised (where necessary), resuspended in 150 μl of PBS and fixed by the dropwise addition of 600 μl of ice-cold 70% ethanol with vortexing. For analysis, cells were washed twice with PBS, exposed to 0.1 mg $ml^{-1}$ RNAse A (Sigma) for 15 min at 37°C and then diluted to $\approx 10^6$ cells $ml^{-1}$. Propidium iodide (1 μg $ml^{-1}$) was added before analysis on a Beckton Dickinson FacsStar. Triplicate samples of 10 000 events each were analysed using the flow cytometer software.

**Figure 1** MTT cytotoxicity dose-response profiles for *R*-$PAF_{16}$ (■), lyso-$PAF_{16}$ (●), HePC (○), ET18-$OCH_3$ (□), SRI 62-834 (▲) and MCP (△) in HT29 cells. The $IC_{50}$ concentrations (dotted line) are those at which MTT dye absorbance was reduced to 50% of control values. Error bars have been omitted as standard deviations of eight replicate wells were routinely below 15% of the mean. The data illustrated are representative of at least four independent experiments and very similar results have also been obtained with HL60 cells (see Table II). Means and standard errors for replicate experiments are given in the text.

### Results

*Structure–cytotoxicity relationships by MTT assay*

Figure 1 shows typical cytotoxicity data in HT29 human colon carcinoma cells, using the MTT assay. Note that there is only a comparatively narrow range over which these agents develop their full effect. Average $IC_{50}$ values for the complete panel of agents, tested against HT29 and HL60 cells, are given in Table II. Despite differences in cell type (carcinoma *vs* leukaemia) and growth characteristics (adherent monolayer *vs* detached single-cell suspension), both cell lines were generally equally sensitive to any given agent. Selected compounds were also evaluated in EMT6 mouse mammary tumour cells. The $IC_{50}$ values for ET18-$OCH_3$ and SRI 62-834 in EMT6 cells were 95 ± 10 and 100 ± 20 μM respectively. $PAF_{18}$ and lyso-$PAF_{18}$ failed to reach the $IC_{50}$ at concentrations up to 150 μM. Thus EMT6 cells were at least 3-fold more resistant to PAF and lyso-PAF and about 30-fold more sensitive to the ATLs.

Table I illustrates the chemical structures for all lipid agents tested. Comparison of individual drug potency against HT29 cells revealed that ATLs, as exemplified by ET18-$OCH_3$ and SRI 62-834, all had $IC_{50}$ values around 2–5 μM. In contrast, the naturally occurring parent compounds of the PAF and lyso-PAF family were much less potent with $IC_{50}$ values in the 40–50 μM range. Interestingly, the synthetic *S* isomer of $PAF_{16}$ was as potent as some of the ATLs. SAH 63-817 was the only compound which proved more toxic in one particular cell line (HT60) than the other (HT29). Intermediate potencies were seen with SAH 62-537 and the

**Table II** Cytotoxicity of lipid agents against HT29 and HL60 cells in the presence and absence of WEB 2086

| | *HT29* | *HL60* | *HT29 +49 μM WEB 2086* | *HL60 +49 μM WEB 2086* |
|---|---|---|---|---|
| $PAF_{18}$ | 45.6 ± 2.4 | 43.8 ± 8.5 | 43.2 ± 4.8 | 50.3 ± 4.8 |
| $PAF_{16}$ (*R*) | 53.6 ± 1.9 | ND | 57.5 ± 4.5 | ND |
| $PAF_{16}$ (*S*) | 8.5/4.0 | ND | ND | ND |
| Lyso-$PAF_{18}$ | 41.4 ± 1.7 | 40.0 ± 4.0 | 45.5 ± 2.2 | 34.6 ± 8.2 |
| Lyso-$PAF_{16}$ | 54.8 ± 3.5 | ND | 56.6 ± 6.9 | ND |
| Arachidonoyl-PAF | 44.3 ± 4.3 | ND | 39.2 ± 5.7 | ND |
| HePC | 15.5 ± 1.4 | 18.1 ± 1.9 | 17.7 ± 0.3 | 16.4 ± 3.4 |
| Methylcarbamyl-PAF | 3.0 ± 0.2 | 3.3 ± 0.1 | 3.2 ± 0.2 | 2.8 ± 0.5 |
| ET18-$OCH_3$ | 2.5 ± 0.3 | 2.6 ± 0.1 | 2.5 ± 0.3 | 2.2 ± 0.3 |
| ET16-$OCH_3$ | 3.6 ± 1.1 | ND | 4.1 ± 1.2 | ND |
| SRI 62-834 | 3.3 ± 0.4 | 2.7 ± 0.1 | 3.1 ± 0.2 | 2.5 ± 0.6 |
| SDZ 266-336 (*S*) | 2.5 ± 0.2 | 3.0 ± 0.3 | 3.0 ± 0.4 | ND |
| SDZ 266-337 (*R*) | 2.1 ± 0.2 | 2.6 ± 0.2 | 2.6 ± 0.4 | ND |
| SAH 62-817 | 35.2 ± 4.9 | 21.3 ± 2.9 | 43.3 ± 4.8 | ND |
| SAH 62-537 | 7.5 ± 1.1 | 9.1 ± 1.1 | 7.5 ± 0.4 | ND |
| SAH 62-990 | 3.1 ± 0.3 | 4.9 ± 0.9 | 4.0 ± 0.2 | ND |
| SAH 63-871 | 2.2 ± 0.3 | 2.2 ± 0.2 | 2.7 ± 0.5 | ND |
| SAH 63-875 | 2.8 ± 0.5 | 2.9/4.2 | 3.6 ± 0.9 | ND |
| BN 52205 | 2.1 ± 0.2 | 3.0 ± 0.2 | ND | ND |
| BN 52207 | 1.4 ± 0.3 | 2.2 ± 0.1 | ND | ND |
| BN 52211 | 2.3 ± 0.6 | 3.7 ± 0.3 | ND | ND |
| Dimyristoyl-PA | > 500 | ND | ND | ND |
| Dipalmitoyl-PA | > 500 | ND | ND | ND |
| Dilauroyl-PA | > 200 | ND | ND | ND |
| Lyso-PA | > 100 | ND | > 100 | ND |

$IC_{50}$ values (μM) are the mean ± standard error of three or more experiments. Where only two experiments were performed, both repeats are given. Suffixes *R* and *S* denote isomerically different compounds. ND, not determined; PA, phosphatidic acid.

alkylphosphocholine HePC. The four different phosphatidic acids all proved comparatively inactive with $IC_{50}$ values in excess of 100 μM.

Also evident from Table II is that none of the complementary ATL stereoisomers (i.e. SAH 63-871 and SAH 63-875 or SDZ 266-336 and SDZ 266-337) showed any marked differences in cytotoxic potency. Differences between the positional isomers (i.e. BN 52205, BN 52207 and BN 52211) were also not statistically significant (*t*-test). Thus molecular stereochemistry clearly does not correlate with anti-tumour activity.

Compounds with differing alkyl chain length (i.e. $C_{16}$ *vs* $C_{18}$) were available for PAF, lyso-PAF and ET18-$OCH_3$. The $C_{18}$ compounds, particularly of PAF and lyso-PAF, were more cytotoxic than their $C_{16}$ counterparts (see Table II). However, the differential was not statistically significant for the more potent anti-tumour agents ET18-$OCH_3$ and ET16-$OCH_3$.

*Lack of toxicity modulation by WEB 2086*

The PAF receptor antagonist WEB 2086 was non-toxic at concentrations exceeding 250 μM. At 49 μM, WEB 2086 had no effect on the cytotoxicities of any of the lipid agents tested in HT29 and HL60 cells (see Table II). These results argue against the involvement of a WEB-sensitive PAF receptor in the cytotoxicity of PAF or its ATL analogues.

*Population growth curve analysis*

Figure 2 illustrates the effect of continuous SRI 62-834 exposure on HL60 cells. Cytostasis occurs at 3–5 μM and population growth arrest persists for ⩾7 days. Similar results were seen in HT29 cells, in agreement with their coordinate MTT data. Again, the dose–response curve is extremely steep for both cell lines with population growth hardly affected at 1 μM, but with almost complete stasis occurring at 2–3 μM.

Figure 3 summarises the comparative effects of ET18-$OCH_3$, SRI 62-834, HePC and $PAF_{16}$ on HL60 cell numbers after 7 days of continuous exposure. Both ET18-$OCH_3$ and SRI 62-834 are equally active with $IC_{50}$ values between 1 and 2 μM. HePC was less active with an $IC_{50}$ of 10–15 μM. $PAF_{16}$ was least potent, effecting half-maximal growth arrest at 40–50 μM. These $IC_{50}$ values agree well with the MTT data (Table II). Complete population growth arrest, i.e. no increase in cell number over the original seeding density, was achieved using ET18-$OCH_3$, SRI 62-834, HePC and $PAF_{16}$ at 3.9, 4.5, 31.6 and ≈125 μM (extrapolated) respectively. Note that these concentrations ($IC_{100}$ values) are only around 2-fold higher than those causing half-maximal growth arrest (Figure 3).

Interestingly, HL60 promyelocytic cells did not differentiate in response to treatment with ATLs at these doses. Cellular differentiation was not evident under the conditions used, as judged by morphology, adhesion and flow cytometric analysis of cell shape and granularity.

We have previously shown that serum concentration affects the *lytic* potency of ET18-$OCH_3$ and SRI 62-834 (Lohmeyer and Workman, 1993). Here, we show that serum concentration also affects the *non-lytic* antiproliferative activity of ATLs. Reducing serum concentrations from 10% to 5.5% and 1% results in markedly slower growth of untreated HT29 cells. Control cell numbers after 7 days were reduced to 55.0% and 9.5% respectively. After continuous exposure to various ATLs, a steep antiproliferative dose–response relationship was evident at all serum levels. The $IC_{50}$ values in 10%, 5.5% and 1% FCS were 1.8, 0.8 and 0.3 μM respectively, confirming the potentiating effects of serum levels on the *non-lytic* antiproliferative activity of ATLs.

*Cell cycle effects of ether lipids*

Population growth curves (e.g. Figure 2) show that cell numbers increase at almost the normal rate for the first 24 h after addition ⩽ 5 μM ATL. Only after that period does a significant slowing of cell proliferation (or an increase in cell death) become apparent. This suggests that the majority of cells are able to proceed through one cell division – or complete the one they are currently in – before arresting. At ⩾10 μM ATL, however, an immediate reduction in cell growth is apparent after 24 h (Figure 2).

Figure 4 shows DNA histograms of HT29 cells treated for 28 h with 2 and 5 μM ET18-$OCH_3$. Progressive reduction of

**Figure 2** Representative population growth curve for HL60 cells exposed to SRI 62-834. Similar results were obtained with other ATLs in HL60 and HT29 cells. SRI 62-834 was added when indicated (arrow) and cell numbers from two replicate wells, counted in triplicate, were averaged for each data point. Error bars have been omitted for clarity. The data illustrated are representative of three independent experiments. ■, control; △, 1 μM SRI 62-834; ▲, 2 μM SRI 62-834; □, 3 μM SRI 62-834; ●, 5 μM SRI 62-834; ○, 10 μM SRI 62-834.

**Figure 3** Comparison of the population growth retarding effects of ET18-$OCH_3$ (■), SRI 62-834 (△), HePC (●) and $PAF_{16}$ (○) in HL60 cells. HL60 cell numbers after 7 days of continuous exposure (see Materials and methods) are expressed as multiples of the initial cell number seeded ($10^4$ cells per well). Concentrations resulted in half-maximal growth arrest ($IC_{50}$) and no net growth above the initial seeding cell number after 7 days are indicated by dotted lines. Similar results were obtained with HT29 cells. Cell numbers from two replicate wells, counted in triplicate, were averaged for each data point. Error bars have been omitted for clarity. The data illustrated are representative of at least two independent experiments.

cells in $G_0$ and $G_1$ phases occurs with a concomitant increase of cells arrested in the $G_2$ or M-phase of the cell cycle (Figure 4a–c). This shows that cells in the $G_2$ or M-phase were unable to proceed through mitosis to the next $G_1$ phase, accumulating instead in the $G_2$/M compartment. The proportion of cells in S-phase was comparatively unchanged by ET18-$OCH_3$ treatment. A large amount of DNA-containing debris was also seen routinely at 5 μM doses (Figure 4c). The amount of fluorescing debris increased from just over 2% in control cells to almost 35% of all 'events' collected at 5 μM ET18-$OCH_3$ (Figure 4c). Thus, ATLs at $\geqslant$5 μM can induce significant cellular fragmentation within 28 h. Interestingly, the debris was non-random in size and scatter characteristics (debris 'peaks' in Figure 4c). This may suggest a controlled process of cell destruction.

Incubating cells for up to 46 h did not markedly effect further shifts in the cell cycle distribution at any given dose of ATL. The major difference observed was a significant increase in the amount of cell debris at $\geqslant$5 μM ATL. Here, up to 48.6% of 'events' constituted DNA-containing debris and cell fragments. At 2 μM ATL, the amount of debris collected was low and similar to that after 28 h. Incubating HT29 cells for 6 h with up to 5 μM ET18-$OCH_3$ did not change normal cell cycle distributions or induce debris formation (not shown). Very similar cell cycle changes were also evident in ATL-treated HL60 cells (Figure 5).

*Reversibility of ATL-induced growth arrest*

The finding that ATLs can induce growth arrest and cellular fragmentation within 28 h led us to investigate the reversibility of ATL-induced damage. After 24 h exposure, HT29 and HL60 cells were hardly affected by $\leqslant$3 μM ET18-$OCH_3$ and regrew as soon as the agent was removed. At 5 μM, the population took longer to recover its normal growth rate, while recovery from 10 μM took between 70 and 90 h. The effects of 48 h exposure are illustrated in Figure 6. HL60 cells treated with 2 μM ET18-$OCH_3$ were able to recover their full growth potential within 24 h – as fast as their 24 h counterparts. However, 3 μM ATL required a recovery time of about 30 h. Between 70 and 90 h were required for 5 μM ET18-$OCH_3$. Progressive population reduction, indicating large-scale cell death, was evident at 5–10 μM and corroborates our earlier flow cytometry results. Thus reversibility was seen, but recovery time was related to both drug concentration and duration of exposure. Similar data were obtained in HT29 cells (not shown).

As mentioned previously, we did not observe any differentiation of HL60 cells as a result of ATL treatment at these moderate doses. Therefore, we are confident that the

**Figure 4** DNA histograms showing the cell cycle arrest of HT29 cells in response to 2 and 5 μM ET18-$OCH_3$. Cells were prepared and analysed as detailed in Materials and methods. (**b** and **c**) Effects of a 28 h exposure to 2 and 5 μM ET18-$OCH_3$ compared with control cells (**a**). These histograms are representative of three independent experiments.

**Figure 5** Changes in the cell cycle distribution of HL60 cells as a result of ET18-$OCH_3$ treatment. Cells were prepared and analysed as detailed in Materials and methods. The proportion of cells in the different cell cycle phases was calculated using the sum of broadened rectangles model available with the flow cytometer. Data points represent the mean percentages of three replicate determinations and the resulting graph is representative of three independent experiments. Error bars have been omitted as the standard deviations of triplicate points were below 10% of the mean. ●, $G_0$/$G_1$; ○, S; ▲, $G_2$/M.

**Figure 6** Representative population growth curves for HL60 cells exposed to ET18-$OCH_3$ for 46 h. Cells were seeded at $10^4$ cells per well as described in Materials and methods and ET18-$OCH_3$ was added when indicated (filled arrow). After 46 h, the drug-containing medium was removed and replaced with fresh, drug-free medium (open arrow). Cell numbers from two replicate wells, counted in triplicate, were averaged for each data point. Error bars have been omitted for clarity. The data illustrated are representative of two independent experiments. Similar data were obtained for HT29 cells. ■, control; □, 2 μM ET18-$OCH_3$; ▲, 3 μM ET18-$OCH_3$; ○, 5 μM ET18-$OCH_3$; ●, 10 μM ET18-$OCH_3$.

observed 'regrowth' did not result from the proliferation of a small non-differentiated population.

*Effects on clonal growth*

Figure 7 shows typical effects of ET18-$OCH_3$, lyso-PAF and methylcarbamyl-PAF (MCP) on HT29 cell cloning. Lyso-PAF markedly increased relative plating efficiency by up to 21% (2.3-fold control) and over a wide range of concentrations from 5 nM up to 5 $\mu$M (Figure 7). ET18-$OCH_3$ also gave significant stimulation (up to 2.5-fold control) of clonal growth, while MCP and SRI 62-834 (not shown) gave only comparatively moderate stimulation (1.2 to 1.5-fold control values) at $\approx$0.1 $\mu$M (Figure 7). Neither racemic PAF nor its *R* or *S* isomers affected clonal growth up to 5 $\mu$M (not shown). At high lipid concentrations (generally $\geqslant 2\,\mu$M), a dose-dependent reduction in colony counts was noted. Colony size did not appear to be affected as a result of lipid exposure.

*Membrane-damaging effects*

Exposing HT29 cells to 23 $\mu$M ($\approx 10 \times$ MTT $IC_{50}$) ET18-$OCH_3$ or SRI 62-834 for up to 60 min had no effect on trypan blue exclusion. In 10% FCS, >98% of cells remained 'viable'. $PAF_{18}$ and lyso-$PAF_{18}$ had no immediately lytic effect in HT29 cells up to their MTT $IC_{50}$ dose. After 60 min at 48 $\mu$M, only 5.4% of cells scored trypan blue positive, had lysed or become detached. At 91 $\mu$M, however, 50.2% of cells had lost membrane integrity within 7 min, increasing to 72.5% after 60 min. Higher concentrations caused immediate lysis of $\geqslant$96% of cells within the first 7 min.

Direct membrane damage was further quantified by $^{51}Cr$ release. The $R_{50}$ values for HL60 and HT29 cell lines are given in Table III. These data confirm that ATLs are not membrane lytic on short-term exposure to concentrations which cause growth inhibition with longer exposures. Moreover, the membrane-damaging potencies of the ATLs were similar to PAF or lyso-PAF. In HT29, the last two compounds both feature $R_{50}$ values <110 $\mu$M, while $R_{50}$ values for most ATLs are between 139 and 145 $\mu$M. Only HePC is significantly less lytic. In HL60 cells the $R_{50}$ values for PAF and lyso-PAF, as well as for ET18-$OCH_3$ and SRI 62-834, were between 105 and 120 $\mu$M. MCP and HePC retained the same degree of potency seen in HT29 cells.

Note that the trypsinisation process necessary with HT29 cells had no detrimental effect on the membrane integrity or the amount of spontaneous $^{51}Cr$ release when applied to HL60 cells (not shown).

The PAF receptor antagonist WEB 2086 caused no increased $^{51}Cr$ release at concentrations up to 200 $\mu$M and also failed to exert a clear modulating effect on the membrane-permeabilising potencies of ATLs (Table III). This shows that the PAF receptor is not involved in the mechanism of action leading to cell lysis.

**Figure 7** The effect of ET18-$OCH_3$ (open bars), lyso-PAF (hatched bars) and MCP (solid bars) on the plating efficiency of HT29 cells. Each column represents colony counts from four replicate plates with standard deviations indicated by error bars. No change in the morphology or size of colonies was apparent. Numerical data for these and other compounds tested are given in the text. The data presented are representative of two (lyso-PAF) and three (ET18-$OCH_3$/MCP) independent experiments.

**Table III** Membrane damage of lipid agents against HL60 and HT29 cells in the presence and absence of WEB 2086

| | *HL60* | *HT29* | *HT29 + 49 µM WEB 2086* |
|---|---|---|---|
| $PAF_{18}$ | 120 ± 3 | 121 ± 14 | 114 ± 14 |
| Lyso-$PAF_{18}$ | 115 ± 24 | 110 ± 11 | 140 ± 10 |
| Methylcarbamyl-PAF | 145/117 | 145 ± 14 | ND |
| HePC | >170 | 190 ± 10 | 184 ± 25 |
| ET18-$OCH_3$ | 115 ± 10 | 139 ± 9 | 128 ± 23 |
| SRI 62-834 | 108 ± 3 | 139 ± 7 | 130 ± 24 |
| WEB 2086 | >200 | >260 | |

$IC_{50}$ values ($\mu$M) are the mean ± standard error of three or more experiments. Where only two experiments were performed, both repeats are given. ND, not determined.

### Discussion

One of the main questions addressed in this study concerns the importance of molecular structure for ATL anti-tumour activity. The great majority of previous structure-activity studies have examined racemic compounds only, ignoring the potential importance of stereochemistry for activity (Berdel *et al.*, 1987; Fromm *et al.*, 1987; Herrmann and Neumann, 1987). Here, we present data for three sets of matched stereoisomers, chosen to reflect different groups of compounds (PAF, the anti-tumour lipid SRI 62-834 and novel compounds SAH 63-871 and 63-875). Further, we examine the importance of molecular configuration using a set of three positional isomers. This work is also the first to systematically separate and recognise three different antiproliferative activities of ATLs, using a combination of cytotoxicity, proliferation and flow cytometric assays. Another novel aspect of this work is our finding that some ATLs can promote mitogenesis at nanomolar doses. Extending previous studies by ourselves and others, we investigate the serum dependence of the *non-lytic* anti-tumour activity of ATLs and the importance of interactions with cellular PAF receptors for cytotoxicity and cell lysis (Andreesen *et al.*, 1982; Fleer *et al.*, 1992; Lohmeyer and Workman, 1992).

The extensive structure–activity data reported here show that the human HT29 colon carcinoma and the HL60 promyelocytic leukaemia cell line were equally sensitive to any given lipid in our panel. This similarity was seen despite the different biological origins (colon epithelial *vs* haematopoietic) and modes of growth (attached monolayer *vs* single-cell suspension). The murine EMT6 mammary carcinoma cell line proved much more resistant to both ATLs and PAF analogues than the human lines. With ATLs, the difference was about 30-fold. With PAF and lyso-PAF, little or no cytostatic effect was seen in EMT6 up to 150 $\mu$M – a concentration 3-fold higher than the $IC_{50}$ in HT29 and HL60 cells. Clearly, EMT6 cells either metabolise and detoxify ATLs more rapidly or lack the specific target(s) responsible for potency in the human lines. Decreased endocytotic activity and/or increased cholesterol levels have also been advanced as a determinant of ATL resistance (Bazill and Dexter, 1990; Diomede *et al.*, 1991; reviewed in Workman, 1991). The effect is unlikely to involve species differences, since other murine cell lines such as WEHI-3B myelomonocytic leukaemia and Meth A fibrosarcoma cells are sensitive to ATLs (Houlihan *et al.*, 1987; Bazill and Dexter, 1990).

To date, three basic key molecular features are recognised for ATL activity: (1) an ether or thioether linked alkyl moiety at the *sn*-1 position of the glycerol backbone; (2) a small substituent at the *sn*-2 position; and (3) a phosphocholine head group at the 3-position of *sn*-glycerol (Fleer *et al.*, 1990; Munder and Westphal, 1990; Vogler *et al.*, 1993*a*). However, these apparent 'rules' are being rapidly eroded as medicinal chemists prepare ever more exotic lipid compounds which nevertheless retain anti-tumour activity (Ishaq *et al.*, 1989; Kasukabe *et al.*, 1990; Marasco *et al.*, 1990; Houlihan *et al.*, 1995).

The most important conclusion from our structure–activity data is that molecular conformation at the *sn*-2 position of the glycerol backbone is *not* an important determinant of activity. Both isomers of SRI 62-834 (i.e. SDZ 226-336 and SDZ 226-337), and SAH 63-871 and SAH 63-875 were equally cytotoxic in spite of their very different conformations. Similarly, the three positional isomers (BN 52205, 52207 and 52211) were found to possess very similar $IC_{50}$ values. The large panel of agents tested also shows that activity is not related to any particular structural feature at the *sn*-2 position. Many very different substituents of greatly varying size proved equally active. Thus, a particular biophysical property of these lipids most likely holds the key to their activity.

Interestingly, the *S* and *R* isomers of $PAF_{16}$ did show a significant difference in cytotoxic potency. The 'synthetic' *S*-PAF was as potent as some of the ATLs, but features the same functional groups as the less toxic *R*-PAF, just in a different conformation. So why do *R*-PAF and its natural analogues not possess potent activity, when molecular structure was so clearly not important for activity of the other isomer pairs? The most likely explanation is the rapid metabolism of natural lipids and the relative metabolic inertness of ATLs. PAF, lyso-PAF and arachidonoyl-PAF are rapidly metabolised by cellular acetylhydrolases (Shen *et al.*, 1987; Nakagawa and Waku, 1989). Although metabolised to some extent (Bishop *et al.*, 1992; Fleer *et al.*, 1992), synthetic lipids such as MCP, ET18-$OCH_3$ and HePC are known to be metabolically stable compounds with long half-lives (Arnold *et al.*, 1978; Hoffman *et al.*, 1986; Breiser *et al.*, 1987). At the present time, we believe the most parsimonious hypothesis to be that ATLs are more potent than natural lipids because of their greater metabolic stability.

The precise molecular mode of action of ATLs remains to be elucidated. The potential involvement of a PAF receptor (Honda *et al.*, 1991) was suggested by the close structural similarity of some ATLs to PAF. ET18-$OCH_3$ and MCP in particular are known to antagonise PAF binding, MCP exhibiting a potent agonist activity (Shen *et al.*, 1987). The triazolodiazepine derivative WEB 2086 potently antagonises the various known PAF effects *in vitro* and *in vivo* (Casals-Stenzel *et al.*, 1987) and has been described as the 'reference PAF antagonist' (Page and Abbott, 1989). Testing a larger panel of agents, we have confirmed our earlier finding (Lohmeyer and Workman, 1992) that WEB 2086 does not affect the cytotoxicity of ATLs or related agents. This conclusion is borne out by other studies showing that the presence of PAF-specific binding sites on neoplastic cells also failed to correlate with their sensitivity to the PAF antagonists (Danhauser-Riedl *et al.*, 1991). Overall, it seems clear that a WEB-sensitive PAF receptor is not involved in the antiproliferative mechanism of ATLs.

Whether ATLs interact in a highly specific way with other cellular proteins is still uncertain (Munder and Westphal, 1990; Berdel, 1991), but this hypothesis becomes increasingly tenuous in the light of our structure–activity data. Given the great structural diversity among active ATLs, it appears unlikely that all of those compounds should be equally active against one or more particular target proteins. Moreover, one might question the significance of various reports of 'specific' interactions of ATLs with a plethora of important cellular proteins such as PKC, phospholipases C and $A_2$, PI-3-kinase, the $Na^+/K^+$-ATPase, $Ca^{2+}$ channels and the epidermal growth receptor (Oishi *et al.*, 1988; Kosano and Takatani, 1989; Powis *et al.*, 1992; Berggren *et al.*, 1993).

All of the proteins known to be affected by ATL exposure are membrane bound or at least membrane associated. Many membrane enzymes and receptors are exquisitely sensitive to their surrounding lipid microenvironment (Epand *et al.*, 1991) and some, e.g. PKC, PLC, phospholipase $A_2$ ($PLA_2$) and PI-3-kinase, require lipid co-factors and/or substrates for optimal activity. All of these proteins are thus potentially susceptible to modulation of their boundary lipids and the general phospholipid environment. Postulating such a comparatively indirect interference as a potential mechanism of action accommodates the multitude of membrane proteins affected and may account for the effectiveness of structurally diverse ATLs. However, other more direct mechanisms of action may well be operating in addition to subtle membrane perturbation.

PAF and the ATLs are known to be membrane-active detergents owing to their amphiphilic nature (Noseda *et al.*, 1989*b*; Sawyer and Andersen, 1989; Kantar *et al.*, 1991). However, an important point to stress is that our experiments show no correlation between the lytic potency of these lipids and their cytostatic potency in long-term proliferation assays. In spite of their very different cytostatic potency, ATLs and PAF were equally lytic. Our results confirm that ATLs do not exert rapid, gross membrane damage until concentrations greatly exceed those required to produce antiproliferative effects on prolonged exposure. Thus, the much speculated upon detergent effect of ATLs (Noseda *et al.*, 1988*b*) is not the cytotoxic lesion at low concentrations of these agents. However, the $IC_{50}$ values for the less potent natural lipids are roughly at the point where membrane damage does become significant ($\leqslant 40\ \mu M$). This suggests that the cytotoxic effect seen with PAF and lyso-PAF in the MTT assays may be due primarily to severe membrane perturbation. These results show that the cytostatic and cytotoxic events during long-term exposure are different from short-term effects at higher doses. This is an important distinction to be borne in mind. We have also confirmed our earlier finding (Workman *et al.*, 1991) that WEB 2086 fails to protect HL60 cells against direct membrane damage by ATLs, using a larger panel of active lipid agents.

Taken together, our various studies suggest that ATLs inhibit cell growth of sensitive HT29 and HL60 cells in a fashion which involves three distinct phases, depending on ATL concentration. (1) At low doses of $1-5\ \mu M$ in serum-containing medium, ATLs effect a gradual cessation of population growth (cytostasis). We show that this growth arrest is predominantly centred on a $G_2/M$ block and a general slowing of cell cycle progression. However, even after 46 h, a sizeable proportion of cells were still found in the $G_1$ and S-phases, indicating that the block in $G_2$ or M is 'leaky'. Using extremely high concentrations of up to $200\ \mu M$ over short periods of time, Principe *et al.* (1992) have reported a similar $G_2/M$ block and some arrest in $G_1$. We have shown that the block in $G_2/M$ is also observed when cells are treated with lower cytostatic concentrations of ATLs.

The ATLs induced cytostasis in HL60 cells without concomitant cellular differentiation. This is unusual in HL60 cells, which will readily differentiate in response to a large variety of agents including retinoic acid, dimethylsulphoxide and other cytotoxic agents (Gallagher *et al.*, 1979; Shoji *et al.*, 1988; Vallari *et al.*, 1988). Some authors have reported differentiation of HL60 cells in response to ATL exposure (Honma *et al.*, 1981, 1991; Vallari *et al.*, 1988; Maurer and Hilgard, 1992), but inhibition of differentiation has also been described in the same model system (Shoji *et al.*, 1988; Kuo *et al.*, 1990; Raynor *et al.*, 1991).

(2) At intermediate concentrations between 5 and $40\ \mu M$, a net reduction of viable cell number is observed (cytotoxicity). The precise mechanism of cell death is not yet known, but our flow cytometry data show dramatically increased debris formation which may be indicative of apoptotic cell death.

Apoptosis has been observed in some leukaemic cell lines, including HL60, in response to challenge with ATLs (Diomede *et al.*, 1993, 1994), but this response is not universal (Morimoto *et al.*, 1991). (3) As concentrations exceed 40 μM, the detergent properties of the ATLs begin to induce direct lytic membrane damage. At these high concentrations, the toxicity differential between ATLs and naturally occurring ether lipids is progressively eroded, with all types of lipid killing cells by rapid membrane lysis.

Our results show that the recovery of cell population growth after ATL treatment is both dose and exposure time related. ATLs are widely believed to integrate into the plasma membrane and possibly other cellular membranes (Hoffman *et al.*, 1986). It was perhaps surprising, therefore, that growth inhibition could be removed by simply replacing the drug-containing medium with fresh, drug-free medium. This suggests a rapid equilibrium between the serum-bound and cell-associated lipid. Cells presumably recover their full growth potential by rapid 'back-exchange' onto serum proteins. This putative recovery mechanism has important implications. To be efficiently exchanged with serum proteins, the majority of ATLs must be associated with the outer leaflet of the plasma membrane. The precise location of ATLs within cell membranes has yet to be established and only little is known to date about related phosphocholine lipids (Sleight and Abanto, 1989; Andreesen *et al.*, 1982; Bazill and Dexter, 1990).

Interestingly, in addition to the antiproliferative effects above, we noted that some ATLs promoted clonogenic colony formation and cell proliferation in MTT assays (not shown). Submicromolar concentrations were seen to stimulate significant increases in colony counts, while concentrations above about 2 μM reduced colony counts dramatically. This potentially mitogenic activity has been mentioned 'in passing' (Hoffman *et al.*, 1984; Mende *et al.*, 1989; Sobottka *et al.*, 1993), but there have been no data to support the hypothesis that some ATLs can stimulate growth at nanomolar doses.

Other evidence for a potentially mitogenic role of ATLs comes from cell signalling experiments. ATLs can elicit lipid-specific calcium elevations in tumour cells (Lazenby *et al.*, 1990; Seewald *et al.*, 1990; Lohmeyer and Workman, 1993). Under certain circumstances, such as at low ATL concentrations, these calcium changes may conceivably serve as mitogenic signals, thus stimulating cell proliferation. On the other hand, it should be noted that certain conventional anti-tumour drugs, such as doxorubicin, can also induce cell proliferation at very low concentrations, probably via a membrane effect (Vichi and Tritton, 1989). It is not clear whether these results are relevant to the clinical use of ATLs, but one possibility is that they may relate especially to the observed immunostimulatory effects.

A factor of definitive clinical relevance is the schedule dependence of ATLs. Our studies highlight the importance of exposure time for the *in vitro* anti-tumour effectiveness of ATLs. We found that with pharmacologically relevant concentrations of ATLs, the cytostatic/cytotoxic effect generally developed after the first 24 h of exposure. This 'induction period' was also noted for other ATL activities, such as the suppression of growth factor signalling (unpublished observation; Berens *et al.*, 1988; Seewald *et al.*, 1990). Moreover, recovery of cell growth following 24 h of exposure was comparatively rapid. Longer exposures (⩾48 h) resulted in more pronounced cytostasis/cytotoxicity, and a much delayed recovery. Under conditions of continuous exposure, 2–3 μM ET18-$OCH_3$ or SRI 62-834 was cytostatic, maintaining growth arrest for over 7 days. The dependence on prolonged exposure for maximal activity has also been commented upon by others (Seewald *et al.*, 1990; Principe *et al.*, 1992). It is possible that the failure of some recent phase I/II clinical trials (Rodriguez *et al.*, 1992; Verweij *et al.*, 1992, 1993) to show activity may lie with the dose schedules used, rather than the efficacy of the drugs *per se*. Clearly, most of the clinical trials to date have failed to match the promise of the *in vitro* and *in vivo* preclinical studies (Lohmeyer and Bittman, 1994; Houlihan *et al.*, 1995). However, success has been reported for local topical administration of HePC in breast cancer (ten Bokkel Huinink *et al.*, 1992; Khayat *et al.*, 1993) and for bone marrow purging, where higher drug levels and longer exposures can be effected (Vogler *et al.*, 1993*b*; Vogler, 1994). Pharmacokinetic monitoring is urgently required to determine whether active concentrations of ATLs can be achieved and maintained clinically. Failure to do this could lead us to discard these mechanistically very interesting agents prematurely.

**Acknowledgements**
We wish to thank Dr Ailsa Campbell and Dr James Clark for their support with the flow cytometry and Dr Ian Freshney for his helpful comments. This work was supported by the UK Medical Research Council (MRC) and the Cancer Research Campaign (CRC). ML is grateful for the award of MRC and CRC Research Studentships and PW acknowledges the award of a CRC Life Fellowship.

**Abbreviations:** ATL(s), anti-tumour lipid(s); HePC, hexadecylphosphocholine; SRI 62-834, (±)-2-{hydroxy[tetrahydro-2-(octadecyloxy) methylfuran-2-yl] methoxyl phosphinyloxy}-*N*,*N*,*N*-trimethylethaniminium hydroxide; ET16-$OCH_3$, 1-*O*-hexadecyl-2-*O*-methyl-*rac*-glycero-3-phosphocholine; ET18-$OCH_3$, 1-*O*-octadecyl-2-*O*-methyl-*rac*-glycero-3-phosphocholine; PAF, 1-*O*-alkyl-2-*O*-acetyl-*sn*-glycero-3-phosphocholine; $PAF_{16}$, 1-*O*-hexadecyl-2-*O*-acetyl-*sn*-glycero-3-phosphocholine; $PAF_{18}$, 1-*O*-octadecyl-2-*O*-acetyl-*sn*-glycero-3-phosphocholine; lyso-PAF, 1-*O*-alkyl-*sn*-glycero-3-phosphocholine; MCP, methylcarbamyl-PAF; MTT, 3-(4,5-dimethylthiazol-2-yl)-2,5-diphenyl tetrazolium bromide; WEB 2086, 3-[4-(chlorophenyl)-9-methyl-6*H*-thieno[3,2-*f*][1,2,4]triazolo-[4,3-*a*][1,4]-diazepin-2-yl]-1-(4-morpholinyl)-1-propanone; PBS, Dulbecco's phosphate-buffered saline; FCS, fetal calf serum; $IC_{50}$, concentration giving half-maximal inhibitory effect; $R_{50}$, concentration resulting in half-maximal radiolabel release.

### References

ANDREESEN R, MODOLELL M, OEPKE GHF, COMMON H, LÖHR GW AND MUNDER PG. (1982). Studies on various parameters influencing leukemic cell destruction by alkyl-lysophospholipids. *Anticancer Res.*, **2**, 95–100.

ARNOLD B, REUTHER R AND WELTZIEN HU. (1978). Distribution and metabolism of synthetic alkyl analogs of lysophosphatidylcholine in mice. *Biochim. Biophys. Acta*, **530**, 47–55.

BAZILL GW AND DEXTER TM. (1990). Role of endocytosis in the action of ether lipids on WEHI-3B, HL60 and FDCP-Mix A4 cells. *Cancer Res.*, **50**, 7505–7512.

BERDEL WE. (1990). Ether lipids and derivatives as investigational anticancer drugs. A brief review. *Onkologie*, **13**, 245–250.

BERDEL WE. (1991). Membrane interactive lipids as experimental anticancer drugs. *Br. J. Cancer*, **64**, 208–211.

BERDEL WE AND MUNDER PG. (1987). Antineoplastic actions of ether lipids related to platelet-activating factor. In *Platelet-Activating Factor and Related Lipid Mediators*, Snyder F (ed.) pp. 449–467. Plenum Press: New York.

BERDEL WE, BAUSERT WR, WELTZIEN HU, MODOLELL ML, WIDMANN KH AND MUNDER PG. (1980). The influence of alkyl-lysophospholipids and lysophospholipid-activated macrophages on the development of metastasis of 3-Lewis lung carcinoma. *Eur. J. Cancer*, **16**, 1199–1204.

BERDEL WE, KORTH R, REICHERT A, HOULIHAN WJ, BICKER U, NOMURA H, VOGLER WR, BENVENISTE J AND RASTETTER J. (1987). Lack of correlation between cytotoxicity of agonists and antagonists of platelet activating factor (Paf-acether) in neoplastic cells and modulation of <$^3$H>-Paf-acether binding to platelets from humans *in vitro*. *Anticancer Res.*, **7**, 1181–1188.

BERENS ME, BAR-SHIRA E, ROSENBLUM ML, NOSEDA A AND MODEST EJ. (1988). Brain tumour cell sensitivity to ether lipid analogs in vitro: effect of treatment period and assay endpoint on interpreted potency (abstract no. 1280). *Proc. Am. Assoc. Cancer Res.*, **29**, 322.

BERGGREN MI, GALLEGOS A, DRESSLER LA, MODEST E AND POWIS G. (1993). Inhibition of the signalling enzyme phosphatidylinositol-3-kinase by antitumor ether lipid analogues. *Cancer Res.*, **53**, 4297–4302.

BERKOVIC D, FLEER EAM, EIBL H AND UNGER C. (1992). Effects of hexadecylphosphocholine on cellular function. *Prog. Exp. Tumor Res.*, **34**, 59–68.

BISHOP FE, DIVE C, FREEMAN S AND GESCHER A. (1992). Is metabolism an important arbiter of anticancer activity of ether lipid metabolism of SRI 62-834 and hexadecylphosphocholine by [31P]-NMR spectroscopy and comparison of their cytotoxicities with those of their metabolites. *Cancer Chemother. Pharmacol.*, **31**, 85–92.

BREISER A, KIM DJ, FLEER EA, DAMENZ W, DRUBE A, BERGER M, NAGEL GA, EIBL H AND UNGER C. (1987). Distribution and metabolism of hexadecylphosphocholine in mice. *Lipids*, **22**, 925–926.

CASALS-STENZEL J, MUACEVIC G AND WEBER KH. (1987). Pharmacological actions of WEB 2086, a new specific antagonist of platelet activating factor. *J. Pharmacol. Exp. Ther.*, **241**, 974–981.

DANHAUSER-RIEDL S, FELIX SB, HOULIHAN WJ, ZAFFERANI M, STEINHAUSER G, OBERBERG D, KALVELAGE H, BUSCH R, RASTETTER J AND BERDEL WE. (1991). Some antagonists of platelet activating factor are cytotoxic for human malignant cell lines. *Cancer Res.*, **51**, 43–48.

DIOMEDE L, BIZZI A, MAGISTRELLI A, MODEST EJ, SALMONA M AND NOSEDA A. (1990). Role of cell cholesterol in modulating antineoplastic ether lipid uptake, membrane effects and cytotoxicity. *Int. J. Cancer*, **46**, 341–346.

DIOMEDE L, PIOVANI B, MODEST EJ, NOSEDA A AND SALMONA M. (1991). Increased ether lipid cytotoxicity by reducing membrane cholesterol content. *Int. J. Cancer*, **49**, 409–413.

DIOMEDE L, BIANCHI R, MODEST EJ, PIOVANI B, BUBBA P AND SALMONA M. (1992). Modulation of ATPase activity by cholesterol and synthetic ether lipids in leukemic cells. *Biochem. Pharmacol.*, **43**, 803–807.

DIOMEDE L, COLOTTA F, PIOVANI B, RE F, MODEST EJ AND SALMONA M. (1993). Induction of apoptosis in human leukemic cells by the ether lipid 1-octadecyl-2-methyl-rac-glycero-3-phosphocholine. A possible basis for selective action. *Int. J. Cancer*, **53**, 124–130.

DIOMEDE L, PIOVANI B, RE F, PRINCIPE P, COLOTTA F, MODEST EJ AND SALMONA M. (1994). The induction of apoptosis is a common feature of the cytotoxic action of ether-linked glycerophospholipids in human leukemic cells. *Int. J. Cancer*, **57**, 645–649.

DIVE C, WATSON JV AND WORKMAN P. (1991). Multiparametric flow cytometry analysis of the modulation of tumor cell membrane permeability by developmental antitumor ether lipid SRI 62-834 in EMT6 mouse mammary tumor and HL-60 human promyelocytic leukemia cells. *Cancer Res.*, **51**, 799–806.

DUMMER R, RÖGER J, VOGT T, BECKER J, HEFNER H, SINDERMANN H AND BURG G. (1992). Topical application of hexadecylphosphocholine in patients with cutaneous lymphomas. *Prog. Exp. Tumor Res.*, **34**, 160–169.

EPAND RM, EPAND RF, LEON BT-C, MENGER FM AND KUO JF. (1991). Evidence for the regulation of the activity of protein kinase C through changes in membrane properties. *Biosci. Rep.*, **11**, 59–64.

FLEER EA, KIM DJ, NAGEL GA, EIBL H AND UNGER C. (1990). Cytotoxic activity of lysophosphatidylcholine analogues on human lymphoma Raji cells. *Onkologie*, **13**, 295–300.

FLEER EAM, BERKOVIC D, UNGER C AND EIBL H. (1992). Cellular uptake and metabolic fate of hexadecylphosphocholine. *Prog. Exp. Tumor Res.*, **34**, 33–46.

FROMM M, BERDEL WE, SCHICK HD, FINK U, PAHLKE W, BICKER U, REICHERT A AND RASTETTER J. (1987). Antineoplastic activity of the thioether lysophospholipid derivative BM 41.440 *in vitro*. *Lipids*, **22**, 916–918.

GALLAGHER R, COLLINS S, TRUJILLO J, McCREDIE K, AHEARN M, TSAI S, METZGAR R, AULAKH G, TING R, RUSCETTI F AND GALLO R. (1979). Characterization of the continuous, differentiating myeloid cell line (HL-60) from a patient with acute promyelocytic leukemia. *Blood*, **54**, 713–733.

GRUNICKE HH. (1991). The cell membrane as a target for cancer chemotherapy. *Eur. J. Cancer*, **27**, 281–284.

HERRMANN DBJ AND NEUMANN HA. (1987). Cytotoxic activity of the thioether phospholipid analogue BM 41.440 in primary human tumor cultures. *Lipids*, **22**, 955–957.

HILGARD P, KAMPHERM E, NOLAN L, POHL J AND REISSMANN T. (1991). Investigation into the immunological effects of miltefosine, a new anticancer agent under development. *J. Cancer Res. Clin. Oncol.*, **117**, 403–408.

HOFFMAN DR, HAJDU J AND SNYDER F. (1984). Cytotoxicity of platelet activating factor and related alkyl-phospholipid analogs in human leukemia cells, polymorphonuclear neutrophils, and skin fibroblasts. *Blood*, **63**, 545–552.

HOFFMAN DR, HOFFMAN LH AND SNYDER F. (1986). Cytotoxicity and metabolism of alkyl phospholipid analogs in neoplastic cells. *Cancer Res.*, **46**, 5803–5809.

HOFFMANN J, UEBERALL F, POSCH L, MALY K, HERMANN DBJ AND GRUNICKE H. (1989). Synergistic enhancement of the antiproliferative activity of cis-Diamminedichloroplatinum(II) by the new ether lipid analogue BM41440, an inhibitor of protein kinase C (abstract no. 2307). *Proc. Am. Assoc. Cancer Res.*, **30**, 580.

HONDA Z, NAKAMURA M, MIKI I, MINAMI M, WATANABE T, SEYAMA Y, OKADO H, TOH H, ITO K, MIYAMOTO T AND SHIMIZU T. (1991). Cloning by functional expression of platelet-activating factor receptor from guinea-pig lung. *Nature*, **349**, 342–346.

HONMA Y, KASUKABE T, HOZUMI M, TSUSHIMA S AND NOMURA H. (1981). Induction of differentiation of cultured human and mouse myeloid leukemia cells by alkyl-lysophospholipids. *Cancer Res.*, **41**, 3211–3216.

HONMA Y, KASUKABE T, HOZUMI M, AKIMOTO H AND NOMURA H. (1991). Induction of differentiation of human myeloid leukemia HL-60 cells by novel nonphosphorus alkyl ether lipids. *Lipids*, **26**, 1445–1449.

HOULIHAN WJ, LEE ML, MUNDER PG, NEMECEK GM, HANDLEY DA, WINSLOW CM, HAPPY J AND JAEGGI C. (1987). Antitumor activity of SRI 62-834, a cyclic ether analog of ET-18-OCH3. *Lipids*, **22**, 884–890.

HOULIHAN WJ, LOHMEYER M, WORKMAN P AND CHEON SH. (1995). Phospholipid antitumor agents. *Med. Res. Rev.*, **15**, 157–223.

ISHAQ KS, CAPOBIANCO M, PIANTADOSI C, NOSEDA A, DANIEL LW AND MODEST EJ. (1989). Synthesis and biological evaluation of ether-linked derivatives of phosphatidylinositol. *Pharmacol. Res.*, **6**, 216–224.

KANTAR A, GIORGI PL, CURATOLA G AND FIORINI R. (1991). Effect of PAF on erythrocyte membrane heterogeneity: a fluorescence study. *Agents Actions*, **32**, 347–350.

KASUKABE T, HONMA Y, HOZUMI M AND NOMURA H. (1990). Inhibition of proliferation and induction of differentiation of human and mouse myeloid leukemia cells by new ethyleneglycol-type nonphosphorus alkyl ether lipids. *Jpn. J. Cancer Res.*, **81**, 807–812.

KHAYAT D, BREAU JL, POUILLART P. MISSET JL, MACHOVER D AND DAVID M. (1993). Miltefosine 6% solution (MIL) as a local treatment in a cutaneous metastases of breast cancer in patients receiving a concomitant systemic therapy. Preliminary results (abstract no. 49). *Proc. Am. Soc. Clin. Oncol.*, **12**, 62.

KOSANO H AND TAKATANI Q. (1989). Inhibition by an alkyl-lysophospholipid of the uptake of epidermal growth factor in human breast cancer cell lines in relation to epidermal growth factor internalization. *Cancer Res.*, **49**, 2868–2870.

KUCERA LS, IYER N, LEAKE E, RABEN A, MODEST EJ, DANIEL LW AND PIANTADOSI C. (1990). Novel membrane-interactive ether lipid analogs that inhibit infectious HIV-1 production and induce defective virus formation. *Aids Res. Hum. Retroviruses*, **6**, 491–501.

KUCERA LS, KRUGNERHIGBY IA, IYER NP, GOFF DH, EDWARDS TV, NEUFELD JA, ANAND IS, PUCKETT S, MORRIS-NATSCHKE SL AND PIANTADOSI C. (1993). Investigations on membrane-active ether lipid analogs that alter functional expression of HIV-1 induced glycoproteins and inhibit pathogenesis. *J. Cell. Biochem.*, **17e**, 16.

KUO JF, ZHENG B, SHOJI M, VOGLER WR AND EIBL H. (1990). Inhibition of protein kinase C, Na,K-ATPase and HL60 cell differentiation by alkylphosphocholine and alkylammonium bromide derivatives. *J. Cancer Res. Clin. Oncol.*, **116S**, 889.

LAZENBY CM, THOMPSON MG AND HICKMAN JA. (1990). Elevation of leukemic cell intracellular calcium by the ether lipid SRI62-834. *Cancer Res.*, **50**, 3327–3330.

LOHMEYER M AND BITTMAN R. (1994). Antitumour ether lipids and alkylphosphocholines. *Drugs Future*, **19**, 1021–1037.

LOHMEYER M AND WORKMAN P. (1992). Lack of enantioselectivity in the *in vitro* antitumour cytotoxicity and membrane-damaging activity of ether lipid SRI 62-834: further evidence for a non-receptor-mediated mechanism of action. *Biochem. Pharmacol.*, **44**, 819-823.

LOHMEYER M AND WORKMAN P. (1993). The role of intracellular free calcium mobilization in the mechanism of action of antitumour ether lipids SRI 62-834 and ET18-OMe. *Biochem. Pharmacol.*, **45**, 77-86.

MANGOLD HK AND WEBER N. (1987). Biosynthesis and biotransformation of ether lipids. *Lipids*, **22**, 789-799.

MARASCO JR CJ, PIANTADOSI C, MEYER KL, MORRIS-NATSCHKE S, ISHAQ KS, SMALL GW AND DANIEL LW. (1990). Synthesis and biological activity of novel quaternary ammonium derivatives of alkylglycerols as potent inhibitors of protein kinase C. *J. Med. Chem.*, **33**, 985-992.

MAURER HR AND HILGARD P. (1992). Induction of tumor cell differentiation by alkylphosphocholines: a new approach for in vitro screening. *Prog. Exp. Tumor Res.*, **34**, 90-97.

MENDE S, TEUSCHER E, WINDECK I, LICHTNOW KH, NUHN P AND BRACHWITZ H. (1989). Zum Einfluß von Alkyllysophospholipiden auf Membranpotential, Teilung und Migration von isolierten Kälberaortenendothelzellen. [The effect of alkyllysophospholipids and membrane potentials, proliferation and migration of isolated calf aorta endothelial cells]. *Pharmazie*, **44**, 713-715.

MEYER KL, MARASCO JR CJ, MORRIS-NATSCHKE SL, ISHAQ KS, PIANTADOSI C AND KUCERA LS. (1991). *In vitro* evaluation of phosphocholine and quarternary ammonium containing lipids as novel anti-HIV agents. *J. Med. Chem.*, **34**, 1377-1383.

MODOLELL M, ANDREESEN R, PAHLKE W, BRUGGER U AND MUNDER PG. (1979). Disturbance of phospholipid metabolism during the selective destruction of tumor cells induced by alkyllysophospholipids. *Cancer Res.*, **39**, 4681-4686.

MORIMOTO H, BROQUET C, PRINCIPE P, MENCIA-HUERTA JM, BRAQUET P AND BONAVIDA B. (1991). Cytotoxic activity of synthetic aza alkyl lysophospholipids against drug sensitive and drug resistant human tumour cell lines. *Anticancer Res.*, **11**, 2223-2230.

MUNDER PG AND WESTPHAL O. (1990). Antitumoral and other biomedical activities of synthetic ether lysophospholipids. In *1939-1989: Fifty Years Progress in Allergy*. Chem. Immunol., Vol. 49, Waksman BH. (ed) pp. 206-235. Karger: Basle.

NAKAGAWA Y AND WAKU K. (1989). The metabolism of glycerophospholipid and its regulation in monocytes and macrophages. *Prog. Lipid Res.*, **28**, 205-243.

NEUMANN HA, LICHTINGHAGEN R, BORCHARDT D AND KISSLER M. (1991). Cytotoxicity of lipid ether ilmofosine in combination with radiotherapy in vitro. *Strahlenther. Onkol.*, **167**, 250-253.

NOSEDA A, BERENS ME, WHITE JG AND MODEST EJ. (1988*a*). *In vitro* antiproliferative activity of combinations of ether lipids analogues and DNA-interactive agents against human tumor cells. *Cancer Res.*, **48**, 1788-1791.

NOSEDA A, GODWIN PL AND MODEST EJ. (1988*b*). Effects of antineoplastic ether lipids on model and biological membranes. *Biochim. Biophys Acta*, **945**, 92-100.

NOSEDA A, WHITE JG, GODWIN PL, JEROME WG AND MODEST EJ. (1989*a*). Membrane damage in leukaemic cells induced by ether and ester lipids: an electron microscopic study. *Exp. Mol. Pathol.*, **50**, 69-83.

NOSEDA A, DIOMEDE L, SALMONA M, GODWIN PL, WHITE JG AND MODEST EJ. (1989*b*). Ether lipid-membrane biophysical interaction. *Cancer Chemother. Pharmacol.*, **24** (suppl. 2), S55.

OISHI K, ZHENG B, WHITE JF, VOGLER WR AND KUO JF. (1988). Inhibition of Na, K-ATPase and sodium pump by anticancer ether lipids and protein kinase C inhibitors ET-18-OCH3 and BM 41.440. *Biochem. Biophys. Res. Commun.*, **157**, 1000-1006.

PAGE C AND ABBOTT A. (1989). PAF: new antagonists, new roles in disease and a major role in reproductive biology. *Trends Pharmacol. Sci.*, **10**, 255-257.

PIGNOL B, CHAUMERON S, COULOMB H, MAISONNET T, VANDAMME B, BROQUET C, MENCIA-HUERTA JM AND BRAQUET P. (1992). Immunomodulatory activity of two new aza alkyl phospholipid antineoplastic drugs. *Anti-Cancer Drugs*, **3**, 599-608.

POWIS G, SEEWALD MJ, GRATAS C, MELDER D, RIEBOW J AND MODEST EJ. (1992). Selective inhibition of phosphatidylinositol phospholipase C by cytotoxic ether lipid analogues. *Cancer Res.*, **52**, 2835-2840.

PRINCIPE P, SIDOTI C AND BRAQUET P. (1992). Tumor cell kinetics following antineoplastic ether phospholipid treatment. *Cancer Res.*, **52**, 2509-2515.

RAYNOR RL, ZHENG B AND KUO JF. (1991). Membrane interactions of amphiphilic polypeptides mastoparan, melittin, polymyxin B, and cardiotoxin. Differential inhibition of protein kinase C, $Ca^{2+}$/calmodulin-dependent protein kinase II and synaptosomal membrane Na,K-ATPase, and $Na^{+}$ pump and differentiation of HL60 cells. *J. Biol. Chem.*, **5**, 2753-2758.

RODRIGUEZ G, WALL J, BURRIS H, HAVLIN K, SHAFFER D, KUHN J, CAGNOLA J, MCPHILLIPS J, HAUSHEER F, WEISS G AND VON HOFF D. (1992). Phase I study of ilmofosine in patients with solid tumors (abstract no. 33). *Ann. Oncol.*, **3** (suppl. 1), 67.

SAWYER DB AND ANDERSEN OS. (1989). Platelet-activating factor is a general membrane perturbant. *Biochim. Biophys. Acta*, **987**, 129-132.

SEEWALD MJ, OLSEN RA, SEHGAL I, MELDER DC, MODEST EJ AND POWIS G. (1990). Inhibition of growth factor-dependent inositol phoshate $Ca^{2+}$ signaling by antitumor ether lipid analogues. *Cancer Res.*, **50**, 4458-4463.

SHEN TY, HWANG S-B, DOEBBER TW AND ROBBINS JC. (1987). The chemical and biological properties of PAF agonists, antagonists, and biosynthetic inhibitors. In *Platelet-Activating Factors and Related Lipid Mediators*, Snyder F. (ed) pp. 153-190. Plenum Press: New York.

SHOJI M, RAYNOR RL, BERDEL WE, VOGLER WR AND KUO JF. (1988). Effects of thioether phospholipid BM 41.440 on protein kinase C and phorbol ester-induced differentiation of human leukemia HL60 and KG-1 cells. *Cancer Res.*, **48**, 6669-6673.

SLEIGHT RG AND ABANTO MN. (1989). Differences in intracellular transport of a fluorescent phosphatidylcholine analog in established cell lines. *J. Cell Sci.*, **93**, 363-374.

SOBOTTKA SB, BERGER MR AND EIBL H. (1993). Structure-activity relationships of four anti-cancer alkylphosphocholine derivatives *in vitro* and *in vivo*. *Int. J. Cancer*, **53**, 418-425.

TALMADGE JE, SCHNEIDER M, LENZ B, PHILLIPS H AND LONG C. (1987). Immunomodulatory and therapeutic properties of alkyl lysophospholipids in mice. *Lipids*, **22**, 871-877.

TEN BOKKEL HUININK WW, SCHORNAGEL A, HILTON A, SOMERS R AND BARTELINK H. (1992). Topical application of miltefosine against skin metastases of breast cancer (abstract no. 39). *Proc. Am. Soc. Clin. Oncol.*, **11**, 53.

ÜBERALL F, OBERHUBER H, MALY K, ZAKNUN J, DEMUTH L AND GRUNICKE HH. (1991). Hexadecylphosphocholine inhibits inositol phosphate formation and protein kinase C activity. *Cancer Res.*, **51**, 807-812.

UNGER C, SINDERMANN H, PEUKERT M, HILGARD P, ENGEL J AND EIBL H. (1992). Hexadecylphosphocholine in the topical treatment of skin metastases in breast cancer patients. *Prog. Exp. Tumor Res.*, **34**, 153-159.

VALLARI DS, SMITH ZL AND SNYDER F. (1988). HL-60 cells become resistant towards antitumor ether-linked phospholipids following differentiation into a granulocytic form. *Biochem. Biophys. Res. Commun.*, **156**, 1-8.

VERWEIJ J, GANDIA D, PLANTING AST, STOTER G AND ARMAND JP. (1992). Phase II study of hexadecylphosphocholine (HePC) in metastatic squamous cell cancer of the head and neck (abstract no. 29). *Ann. Oncol.*, **3** (suppl. 1), 66.

VERWEIJ J, KRZEMIENIECKI K, KOK T, POVEDA A, VAN POTTELSBERGHE C, VAN GLABBEKE M AND MOURIDSEN H. (1993). Phase II study of miltefosine (hexadecylphosphocholine) in advanced soft tissue sarcomas of the adult - an EORTC Soft Tissue and Bone Sarcoma Group study. *Eur. J. Cancer*, **29A**, 208-209.

VICHI P AND TRITTON TR. (1989). Stimulation of growth in human and murine cells by adriamycin. *Cancer Res.*, **49**, 2679-2682.

VOGLER WR. (1994). Bone marrow purging in acute leukemia with alkyl-lysophospholipids: a new family of anticancer drugs. *Leuk. Lymphoma*, **13**, 53-60.

VOGLER WR, OLSON AC, HAJDU J, SHOJI M, RAYNOR R AND KUO JF. (1993*a*). Structure-function relationships of alkyllysophospholipids analogs in selective antitumor activity. *Lipids*, **28**, 511-516.

VOGLER WR, BERDEL WE, HEFFNER LT, WINTON EF, GELLER RB, HOLLAND HK, HILLYER CD, SEAY TE, WINGARD JR AND SARAL R. (1993*b*). Comparison of remission duration, marrow recovery and time of harvest in acute leukemia patients undergoing autologous marrow transplant with edelfosine purged marrow (abstract no. 1032). *Proc. Am. Soc. Clin. Oncol.*, **12**, 313.

WORKMAN P. (1991). Antitumor ether lipids: endocytosis as a determinant of cellular sensitivity? *Cancer Cells*, **3**, 315-317.

WORKMAN P, DONALDSON J AND LOHMEYER M. (1991). Platelet-activating factor (PAF) antagonist WEB 2086 does not modulate the cytotoxicity of PAF or antitumor alkyl lysophospholipids ET-18-O-Methyl and SRI 62-834 in HL-60 promyelocytic leukaemia cells. *Biochem. Pharmacol.*, **41**, 319-322.